# Nectin-4 expression in upper and lower tract urothelial carcinoma: correlation with early-stage disease and prognostic relevance

**DOI:** 10.1007/s00428-025-04164-9

**Published:** 2025-06-27

**Authors:** Go Kobayashi, Yohei Sekino, Tetsutaro Hayashi, Hikaru Nakahara, Kazuma Yukihiro, Kohei Kobatake, Hiroyuki Kitano, Keisuke Goto, Hiroaki Niitsu, Takao Hinoi, Kazuhiro Sentani, Nobuyuki Hinata

**Affiliations:** 1 Department of Molecular Pathology, Graduate School of Biomedical and Health Sciences, Hiroshima University, Hiroshima, Japan; 2 Department of Urology, Graduate School of Biomedical and Health Sciences, Hiroshima University, Hiroshima, Japan; 3 Department of Clinical and Molecular Genetics, Hiroshima University Hospital, Hiroshima, Japan

**Keywords:** Nectin-4, Upper tract urothelial carcinoma, Bladder urothelial carcinoma, Clinicopathological significance, Immunohistochemistry

## Abstract

Nectin-4 is a recognized therapeutic target in advanced or metastatic urothelial carcinoma (UC), with the antibody‒drug conjugate enfortumab vedotin (EV) already approved for clinical use. However, the clinicopathological significance of Nectin-4 expression in UC remains controversial. To address this, we analyzed the expression and distribution of Nectin-4 in 147 upper tract UC (UTUC) and 93 bladder UC (BLCA) tissue samples using immunohistochemistry (IHC). High Nectin-4 expression was observed in cancerous regions, with both membranous and cytoplasmic staining patterns identified. Nectin-4 expression was detected in 84% of UTUC and 83% of BLCA cases and was associated with papillary morphology, low tumor grade, early pathological T stage, and a favorable prognosis. Compared with membranous staining, cytoplasmic staining was more frequently observed in advanced-stage UC. In UTUC, Nectin-4 expression correlated with UPK3 and GATA3 expression and inversely associated with PD-L1 and CK5/6. A combined model incorporating Nectin-4, UPK3, GATA3, and PD-L1 expression demonstrated high predictive performance for the patient prognosis. In silico analyses confirmed the association of *NECTIN4* expression with a favorable prognosis in both UTUC and BLCA patients using the same cutoff values as the Hiroshima cohort. Bioinformatics analysis of RNA-Seq datasets revealed that the *NECTIN4*-high group was significantly associated with pathways such as oxidative phosphorylation, lipid metabolism, and chemical carcinogenesis, whereas the *NECTIN4*-low group was linked to epithelial‒mesenchymal transition (EMT). These findings underscore the clinical value of assessing Nectin-4 protein expression and localization through IHC, which may inform treatment strategies and surveillance protocols for UTUC and BLCA patients.

**Supplementary Information:**

The online version contains supplementary material available at 10.1007/s00428-025-04164-9.

## Introduction

Urothelial carcinoma (UC) is a common malignancy, with the majority of cases occurring as urinary bladder UC (BLCA). Upper tract UC (UTUC), however, is relatively rare, accounting for approximately 5–10% of all urothelial tumors [1]. Although the tumor morphologies of UTUC and BLCA are similar, their genotypes, phenotypes, clinical behaviors, and prognoses differ significantly [2, 3]. Unlike BLCA, UTUC presents as an invasive disease at diagnosis in 60% of cases and is a poor prognostic factor [4]. Therefore, identifying reliable prognostic predictors is crucial for clinical decision-making, particularly in UTUC. Currently, major clinicopathological parameters, including stage, grade, and morphology, are recognized as prognostic factors [1, 5]. However, their predictive accuracy is limited due to individual variations. Recent studies have highlighted the prognostic significance of several biomarkers in UTUC [6–13]. Despite these advancements, clinical biomarkers capable of effectively predicting the patient prognosis and therapeutic response are lacking.

The transmembrane protein Nectin-4, encoded by the *NECTIN4* gene (also known as PVRL4), plays a crucial role in the formation and maintenance of cell–cell adhesion [14]. Nectin-4 is also involved in regulating various intracellular processes, including signal transduction pathways, cell proliferation, differentiation, and apoptosis [15]. It is overexpressed in several human epithelial malignancies, including those of the bladder, lung, breast, and ovary [15, 16]. In UC, the Nectin-4-targeting antibody‒drug conjugate (ADC) enfortumab vedotin (EV) has been approved for the treatment of patients with locally advanced or metastatic UC. ADCs combine the target specificity of monoclonal antibodies with the cytotoxicity of chemotherapy, offering a novel and promising therapeutic strategy [17]. Multiple studies have demonstrated the therapeutic efficacy of EVs in metastatic and advanced UC [18–20]. Although evidence remains limited, recent studies have shown that the therapeutic response of EV appears to be strongly dependent on membranous Nectin-4 expression levels, suggesting a potential predictive role for Nectin-4 in ADC-based treatment [21, 22]. In terms of prognosis, a recent study reported that Nectin-4 expression is a poor prognosis prognostic factor in UTUC [23]. In contrast, other studies have revealed associations between Nectin-4 expression and early-stage disease or luminal marker expression in BLCA [24–26]. Thus, the clinicopathological characteristics and prognostic significance of Nectin-4 in UC remain controversial. Furthermore, the relationship between Nectin-4 localization patterns and the prognosis is not well understood. Finally, the key signaling pathways and molecular mechanisms underlying UTUC with or without *NECTIN4* expression remain largely unexplored.

We investigated the distribution and clinicopathological significance of Nectin-4 expression and its impact on the patient prognosis in UC using surgical tissue samples from UTUC and BLCA patients. We also evaluated the associations between Nectin-4 expression and key cancer-related molecules. Furthermore, we assessed the prognostic significance of Nectin-4 in combination with other markers.

Additionally, we conducted further investigations using public databases. By leveraging RNA-seq data from UTUC and BLCA, we utilized a bioinformatics approach to explore differences in gene functions between patients with high and low Nectin-4 expression.

## Materials and methods

### Patients with UTUC

The medical records of patients who underwent radical nephroureterectomy for unilateral UTUC at Hiroshima University Hospital between April 1999 and May 2019 were retrospectively reviewed. Patients who received neoadjuvant chemotherapy were excluded from this study. Pathology specimens were examined and re-reviewed for staging according to the 8th edition of the American Joint Committee on Cancer/Union for International Cancer Control (AJCC/UICC) TNM classification (2017). We used the 2004 WHO/ISUP 2-tier grading system to evaluate the tumor grade. The study endpoints were CSS and PFS, with progression defined as lymph node relapse or distant metastasis (excluding bladder cancer recurrence or contralateral UTUC). Follow-up included urinalysis and chest-abdomen-pelvis CT every 3–6 months for at least five years, per physician preference.

Patients who were diagnosed with BC and treated with radical cystectomy (RC) at Hiroshima University Hospital or affiliated hospitals in 1995–2015 were included if they did not undergo neoadjuvant chemotherapy. We collected tumor samples from 93 patients who had undergone RC. Tumor staging and TNM pathological classification were conducted according to the 1973 WHO International Society of Urological Pathology Consensus classification and the UICC TNM Classification of Malignant Tumors (7th edition).

### Immunohistochemistry (IHC)

IHC was performed on one or two representative tumor blocks, including the tumor center and invasive front, according to the manufacturer's protocol. The IHC protocol has been previously described [13]. A rabbit monoclonal anti-Nectin-4 antibody ab192033 (dilution 1:1000, Abcam) was used. Nectin-4 expression was primarily observed in the membranes of UC cells, although cytoplasmic staining was also evident. Therefore, Nectin-4 positivity was defined as detectable staining in either the membrane or cytoplasm of tumor cells. Among the Nectin-4–positive cases, tumors were further classified into two expression patterns: membranous (M), in which staining was predominantly observed on the cell membrane, and cytoplasmic (C), in which staining was mainly cytoplasm. Additionally, the clinicopathological significance of these expression patterns was evaluated separately in the UTUC and BLCA cohorts. The evaluation of Ki-67, PD‐L1, CD8, HER2, EGFR, FGFR3, p53, GATA3, UPK3 and CK5/6 expression has also been previously described [17–21] (Table 1).

**Table 1:** Relationship between immunohistochemical positivity for Nectin-4 and clinicopathological parameters in 147 cases of upper tract urothelial carcinoma

	Nectin-4 expression		*P* value
	High (%)	Low (%)	
Age			
< 73 years (*n = *74)	64 (86%)	10 (14%)	0.3805
≥ 73 years (*n = *73)	59 (81%)	14 (19%)	
Sex			
Female (*n = *33)	29 (88%)	4 (12%)	0.5968
Man (*n = *114)	94 (82%)	20 (18%)	
Lateralization			
Right (*n = *66)	52 (79%)	14 (21%)	0.1805
Left (*n = *81)	71 (88%)	10 (12%)	
Location			
Renal pelvis (*n = *69)	60 (87%)	9 (13%)	0.3744
Ureter (*n = *78)	63 (81%)	15 (19%)	
Morphology			
Papillary (*n = *83)	79 (95%)	4 (5%)	** < 0.0001**
Nodular/Flat (*n = *64)	44 (69%)	20 (31%)	
Histological grade			
Low grade (*n = *64)	62 (97%)	2 (3%)	** < 0.0001**
High grade (*n = *83)	61 (73%)	22 (27%)	
Pathological T stage			
pTa/is/1 (*n = *77)	74 (96%)	3 (4%)	** < 0.0001**
pT2/3/4 (*n = *70)	49 (70%)	21 (30%)	

*P* values were calculated with Fisher’s exact test

Bold values show the statistical significance at the *P* < 0.05 level

### In silico analysis

RNA sequence data and clinical data, including 158 UTUC and 8 normal tissues from the study by Fujii et al. [27], were downloaded from the European Genome-phenome Archive (EGA; https://ega-archive.org/). Additionally, RNA-Seq and a clinical dataset of BLCA (*n = *407) tissue samples were obtained from The Cancer Genome Atlas (TCGA) on cBioPortal (https://www.cbioportal.org) [28]. In the TCGA BLCA dataset, tumor morphology data were unavailable for 6 cases, histological grade information was missing for 3 cases, and pathological stage data were missing for 2 cases. Additionally, 14 cases with an unknown cause of death were excluded from the CSS evaluation. Survival and bioinformatics analyses were conducted using these datasets. Details of bioinformatics analysis are provided in Supplementary Text 1.

### Ectopic NECTIN4 expression in UC cells

For ectopic *NECTIN4* expression in UMUC-3 cells, the human *NECTIN4* ORF was cloned and inserted into the lentiviral vector pCDH-MSCV-MCS-EF1-GFP-T2A-Puro (RRID: Addgene_102325); an empty vector served as the control. The construct was cotransfected with helper plasmids (PMDLg/pRRE, pRSV-Rev [RRID: Addgene_12253], and pVSV-G [RRID: Addgene_138479]) into Lenti-X 293 T cells (Takara) using Lipofectamine 2000 (Thermo Fisher). After 48 h, the viral supernatant was collected, filtered, concentrated (Lenti-X concentrator, Takara), and used to infect target cells overnight. GFP-positive cells were sorted using a BD FACSAria II (BD Biosciences). Western blotting was performed as previously described [29]. Antibody information is listed in Supplementary Table 1.

### Statistical analysis

All statistical analyses were performed using SPSS (SPSS Inc., Chicago, IL, USA). Correlations between clinicopathological parameters and Nectin-4 expression were analyzed using Fisher’s exact test. Kaplan–Meier survival curves were constructed for Nectin-4-high and Nectin-4-low patients, and survival was compared. Statistical significance of differences between survival curves was tested by a log-rank test. Univariate and multivariate Cox proportional hazards regression analyses were performed to evaluate associations of clinical covariates or various molecules with survival. Expression was compared between two groups by the Wilcoxon signed‐rank test. Least absolute shrinkage and selection operator (LASSO) Cox regression analysis was used to establish a prognostic model. *P* values of < 0.05 were considered statistically significant.

The receiver operating characteristic (ROC) curve, C-index curve, support vector machine (SVM) and Random Forest classifier were also constructed by installing Python 3.0 on Jupyter Notebook (ver. 6.3.0). The codes used for these analyses are presented in Supplementary Data 1 or described previously [13].

## Results

### Expression and distribution of Nectin-4 in UTUC tissues

**Fig. 1:**
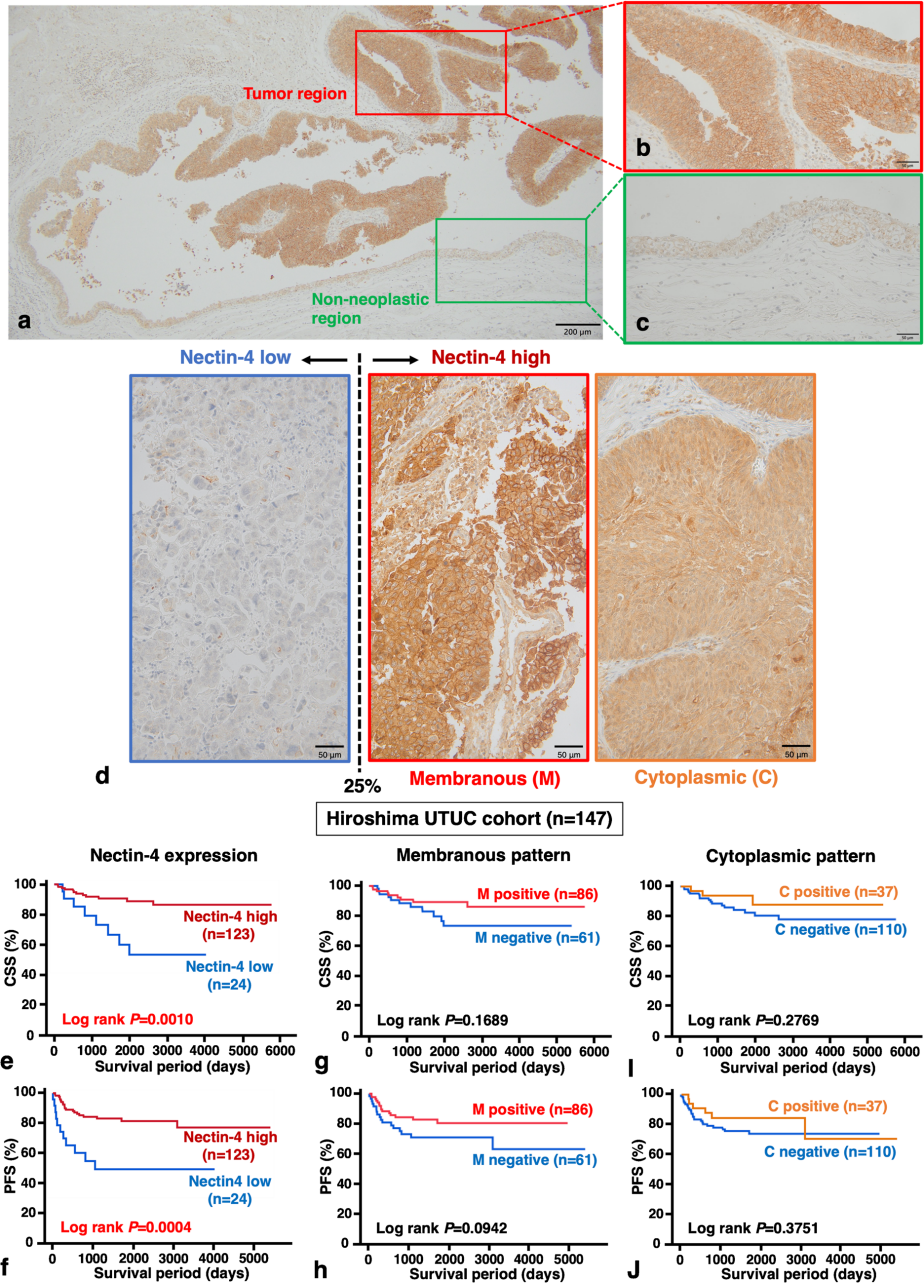
Immunohistochemical analysis of Nectin-4 expression in urothelial carcinoma (UC) tissues. **a** Representative images of Nectin-4 staining in cancerous and adjacent noncancerous regions. The scale bar indicates 200 µm. **b** High-magnification image of Nectin-4 expression in the cancerous region. **c** High-magnification image of Nectin-4 expression in a noncancerous region. **d** Representative images of Nectin-4-high/low and membranous (M)/cytoplasmic (C) staining. **b**-**d** Scale bar indicates 50 µm. **e**-**j** Survival analysis. Kaplan–Meier plots illustrating (**e**) cancer-specific survival (CSS) and (**f**) progression-free survival (PFS) of upper tract urothelial cancer (UTUC) patients stratified by high and low Nectin-4 expression in the Hiroshima cohort. **g**,** h** CSS and PFS stratified by predominant membranous (M) expression. **i**,** j** CSS and PFS stratified by predominant cytoplasmic (C) expression

We first performed IHC to evaluate the expression and distribution of Nectin-4 in UC tissues. Similar expression and distribution patterns were observed in both UTUC and BLCA. In nonneoplastic urothelium, Nectin-4 staining was weak or absent (Fig. 1A, B), whereas high Nectin-4 expression was detected in tumor regions (Fig. 1A, C). Tumors were considered Nectin-4-positive when > 25% of tumor cells were stained, and Nectin-4 positivity was further classified into M and C localization patterns (Fig. 1D).

We evaluated Nectin-4 expression by IHC in 147 UTUC tissue samples. UTUC samples are advantageous for correlating molecular expression with clinicopathological factors across various stages of disease treated by radical nephroureterectomy, without the potential influence of transurethral resection (TUR). Nectin-4 positivity was detected in 123 (84%) of 147 UTUC patients, and its expression was significantly associated with papillary morphology (*p* < 0.0001), low tumor grade (*p* < 0.0001), and early pathological T stage (*p* < 0.0001). Kaplan–Meier analyses revealed that Nectin-4 expression was significantly associated with improved CSS (*p* = 0.0010, Fig. 1E) and PFS (*p* = 0.0004, Fig. 1F). Among the 123 Nectin-4–positive cases, 86 exhibited M staining and 37 exhibited C staining. M-positive cases were associated with papillary morphology (*p* = 0.0014), low tumor grade (*p* = 0.0295), and early pathological T stage (*p* = 0.0004), while C-positive cases were associated with renal pelvic location (*p* = 0.0044) (Table S2−3). The population of C staining in advanced-stage UTUC was greater than in early-stage UTUC (Fig. S1A). Although separate analysis of M or C staining did not reveal statistically significant differences in prognosis, patients with predominant membranous Nectin-4 expression exhibited a trend toward better outcomes in UTUC (Fig. 1G-J). In addition, we analyzed the prognostic effect of Nectin-4 in the pTa/is/1 (non-muscle-invasive) and pT2/3/4 (muscle-invasive) UTUC, respectively. While not statistical significance, a favorable trend was observed in muscle-invasive UTUC cases (Fig. S1B, C).

We further investigated the relationship between Nectin-4 expression and various cancer-related molecules in 147 UTUC patients. Nectin-4 expression was positively associated with UPK3 and GATA3 expression, and inversely associated with PD-L1 expression in tumor-infiltrating lymphocytes and CK5/6 (Table 2).

**Table 2:** Relationship between the expression of Nectin-4 and various cancer-related molecules in 147 cases of upper tract urothelial carcinoma

	Nectin-4 expression		P value
	High (%)	Low	
Ki-67			
Positive (> 20%)	34 (79%)	9 (21%)	0.3361
Negative (≤ 20%)	89 (86%)	15 (14%)	
PD-L1 in TCs			
Positive	14 (70%)	6 (30%)	0.1000
Negative	109 (86%)	18 (14%)	
PD-L1 in TILs			
Positive	33 (70%)	14 (30%)	**0.0039**
Negative	90 (90%)	10 (10%)	
CD8 in TCs			
Positive	43 (86%)	7 (14%)	0.6451
Negative	80 (82%)	17 (18%)	
HER2			
Positive	23 (85%)	4 (14%)	1.0000
Negative	100 (83%)	20 (17%)	
EGFR			
Positive	13 (72%)	5 (26%)	0.1765
Negative	110 (85%)	19 (15%)	
FGFR3			
Positive	42 (93%)	3 (7%)	0.0507
Negative	81 (80%)	21 (20%)	
p53			
Positive	35 (76%)	11 (24%)	0.0994
Negative	88 (87%)	13 (13%)	
GATA3			
Positive	114 (88%)	15 (12%)	**0.0004**
Negative	9 (50%)	9 (50%)	
UPK3			
Positive	49 (96%)	2 (4%)	**0.0022**
Negative	74 (77%)	22 (23%)	
CK 5/6			
Positive	22 (69%)	10 (31%)	**0.0150**
Negative	101 (88%)	14 (12%)	

*P* values were calculated with Fisher’s exact test

Bold values show the statistical significance at the *P* < 0.05 level

Abbreviations: *PD-L1* programmed death ligand 1, *TCs* tumor cells, *TILs* tumor-infiltrating lymphocyte, *HER2* human epidermal growth factor receptor type 2, *EGFR* epidermal growth factor receptor, *FGFR3* fibroblast growth factor receptor 3

### Prognostic analysis of Nectin-4 combined with various markers

High Nectin-4 expression was associated with luminal markers and a favorable prognosis, but its value in predicting other factors was limited. To increase prognostic accuracy, we developed a model integrating biomarkers from Table 2 using LASSO Cox regression. The optimal lambda identified four key factors: Nectin-4, PD-L1 in TC, UPK3, and GATA3 (Fig. S2A, B).

The immunoscore (IS) was calculated as follows:

$${\boldsymbol{N}}{\boldsymbol{e}}{\boldsymbol{c}}{\boldsymbol{t}}{\boldsymbol{i}}{\boldsymbol{n}}-4\,\boldsymbol{ }{\boldsymbol{e}}{\boldsymbol{t}}\boldsymbol{ }{\boldsymbol{a}}{\boldsymbol{l}}.\,\boldsymbol{ }{\boldsymbol{I}}{\boldsymbol{S}}\boldsymbol{ }=\boldsymbol{ }(-0.601549\boldsymbol{ }\times \boldsymbol{ }{\boldsymbol{N}}{\boldsymbol{e}}{\boldsymbol{c}}{\boldsymbol{t}}{\boldsymbol{i}}{\boldsymbol{n}}-4\boldsymbol{\%})\boldsymbol{ }+\boldsymbol{ }(0.6624851\boldsymbol{ }\times \boldsymbol{ }{\boldsymbol{P}}{\boldsymbol{D}}-{\boldsymbol{L}}1\boldsymbol{ }{\boldsymbol{i}}{\boldsymbol{n}}\boldsymbol{ }{\boldsymbol{T}}{\boldsymbol{C}})\boldsymbol{ }+\boldsymbol{ }(-0.721728\boldsymbol{ }\times \boldsymbol{ }{\boldsymbol{U}}{\boldsymbol{P}}{\boldsymbol{K}}3)\boldsymbol{ }+\boldsymbol{ }(-0.177706\boldsymbol{ }\times \boldsymbol{ }{\boldsymbol{G}}{\boldsymbol{A}}{\boldsymbol{T}}{\boldsymbol{A}}3).$$

Using the median as a cutoff value, 147 UTUC patients were classified into high- and low-IS groups, with high-IS significantly associated with worse CSS (*p* < 0.0001, Fig. S2C) and PFS (*p* < 0.0001, Fig. S2D). Additionally, stratification by stage demonstrated that high IS was significantly associated with worse CSS (*p* = 0.0212) and PFS (*p* = 0.0190) in pT2/3/4 UTUC (Fig. S2E-H). Cox analysis confirmed that the IS was an independent predictor for PFS and a marginally significant predictor for CSS (Table 3).

**Table 3:** Univariate and multivariate Cox proportional hazards analyses of cancer-specific survival in 147 cases of upper tract urothelial carcinoma

	Cancer specific survival				Progression free survival			
	Univariate analysis		Multivariate analysis		Univariate analysis		Multivariate analysis	
	HR (95% CI)	*P*	HR (95% CI)	*P*	HR (95% CI)	*P*	HR (95% CI)	*P*
Morphology								
Papillary	1 (Reference)	**0.0008**	1 (Reference)	0.0955	1 (Reference)	< 0.0001	1 (Reference)	**0.0245**
Nodular/Flat	6.59 (2.41–23.05)		2.76 (0.92–10.51)		6.14 (2.80–15.42)		2.95 (1.21–8.13)	
Grade								
Low grade	1 (Reference)	**0.0145**	1 (Reference)	0.5987	1 (Reference)	0.0011	1 (Reference)	0.6677
High grade	3.93 (1.44–13.75)		0.72 (0.23–2.79)		4.37 (1.92–11.77)		1.25 (0.48–3.75)	
pT stage								
pTa/is/1	1 (Reference)	** < 0.0001**	1 (Reference)	**0.0168**	1 (Reference)	< 0.0001	1 (Reference)	**0.0312**
pT2/3/4	25.22 (5.23–453.13)		13.74 (2.31–264.26)		7.79 (3.26–23.02)		3.36 (1.19–11.19)	
Nectin-4 et al. IS								
high	1 (Reference)	**0.0002**	1 (Reference)	0.0678	1 (Reference)	< 0.0001	1 (Reference)	**0.0407**
low	5.46 (2.26–13.97)		2.41 (0.95–6.45)		4.49 (2.23–9.12)		2.18 (1.04–4.66)	

Abbreviations: *HR*hazard ratio, *CI* confidence interval

ROC and C-index analyses demonstrated the superior prognostic performance of the IS (Fig. S2I–L). Furthermore, integrating the IS into clinicopathological models using SVM and random forest classifiers significantly improved the predictive accuracy for CSS and PFS (Fig. S3). Compared with clinicopathological data alone, a 40:60 training-test split validated the enhanced performance (Fig. S3).

### In silico analysis of *Nectin-4* expression in UTUC

**Fig. 2:**
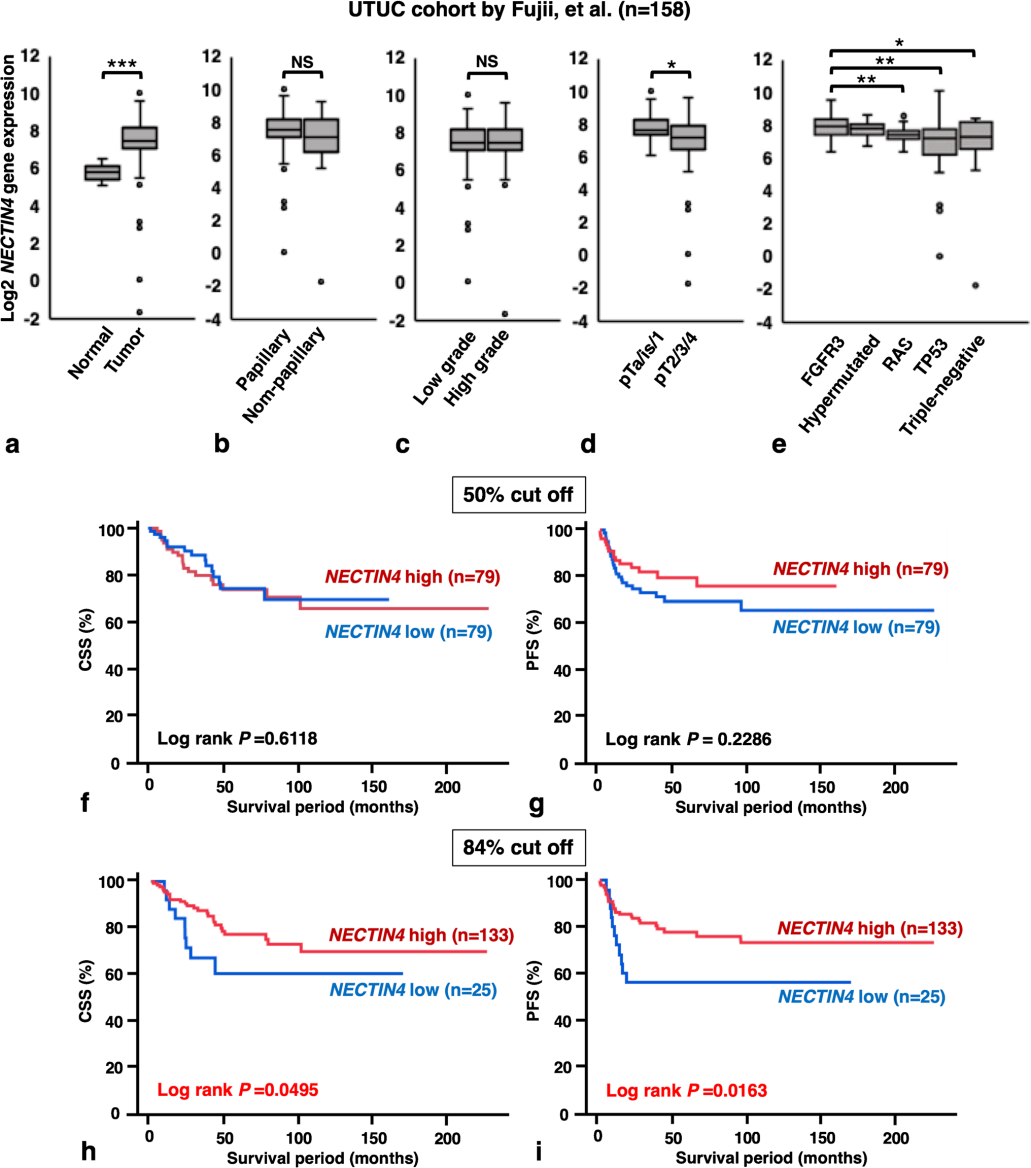
In silico analysis of *NECTIN4* expression in upper tract urothelial carcinoma (UTUC) by Fujii et al. **a** *NECTIN4* expression in normal and tumor tissues. **b**-**e** Association of *NECTIN4* expression with clinicopathological features and mutational subtype. Statistical significance was determined by the Mann‒Whitney U test. **f, g** Kaplan‒Meier plot for cancer-specific survival (CSS) and progression-free survival (PFS) using the median *NECTIN4* expression level as a cutoff value. **h, i** Kaplan‒Meier plot for CSS and PFS using a cutoff value of 84% for *NECTIN4* expression

To validate the IHC findings, we evaluated the relationship between *NECTIN4* expression and several parameters using the public UTUC database reported by Fujii et al. [27]. *NECTIN4* expression was significantly higher in UTUC tissues than in normal urothelium (Fig. 2A). Increased *NECTIN4* expression was observed in patients with lower T stages and the FGFR3 mutational subtype (Fig. 2B–E). Notably, with a cutoff value of 84% (the median expression), while there was no significant difference in the patient prognosis based on *NECTIN4* expression using (Fig. 2F, G), high *NECTIN4* expression was significantly associated with increased CSS (*p* = 0.0495, Fig. 2H) and PFS (*p* = 0.0163, Fig. 2I). This was consistent with findings from the Hiroshima cohort. Furthermore, stratified survival analysis demonstrated that high *NECTIN4* expression was significantly associated with improved PFS in pTa/is/1 cases (*p* = 0.0051, Fig. S4A), whereas no significant association was observed in pT2/3/4 cases (Fig. S4B).

**Fig. 3:**
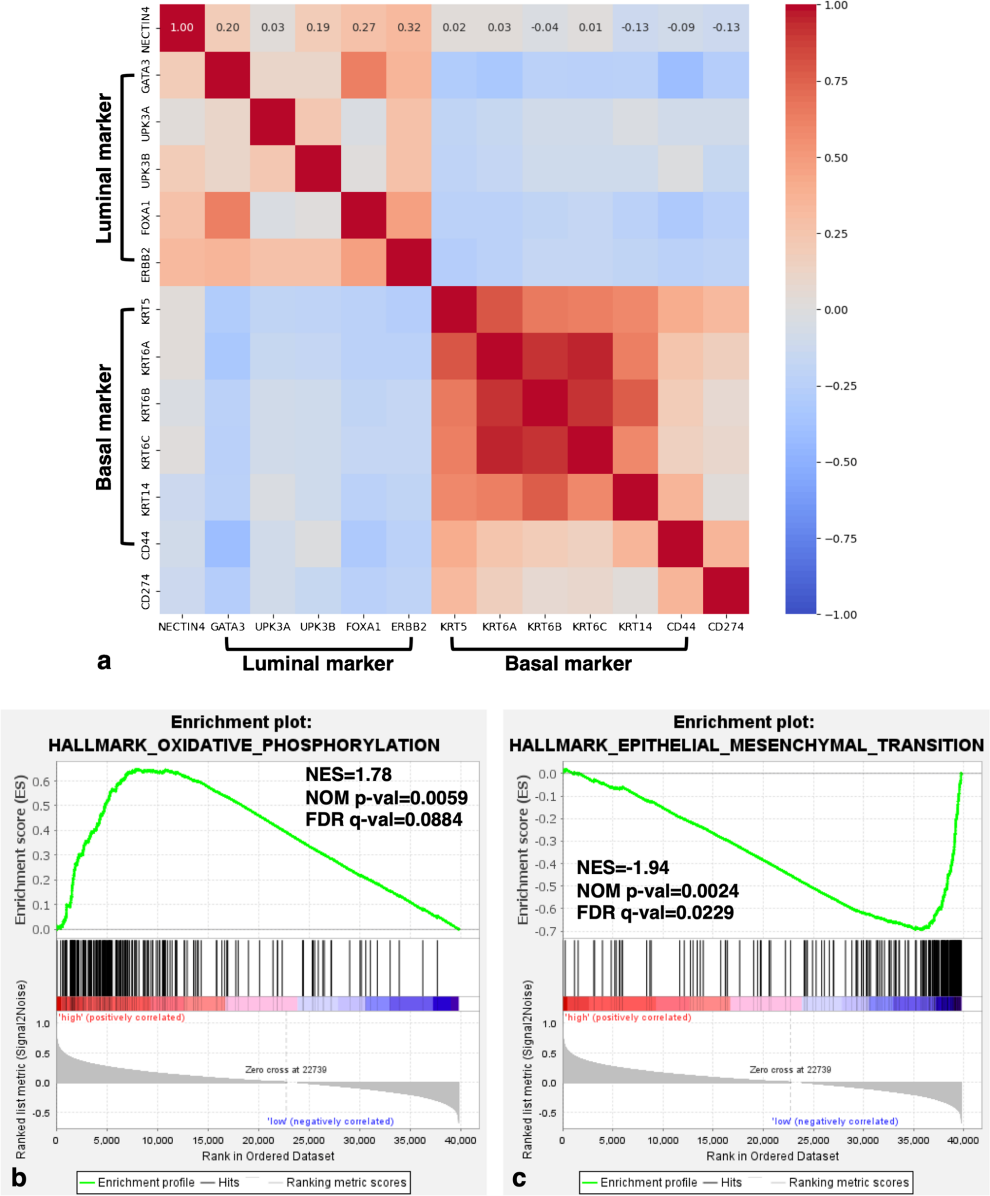
Analysis of the upper tract urothelial carcinoma RNA-seq dataset. **a** Correlation analysis between *NECTIN4*, luminal (*GATA3*, *UPK3A*, *UPK3B*, *FOXA1*, and *ERBB2*) and basal (*KRT5*, *KRT6A*, *KRT6B*, *KRT6C*, *KRT14*, and *CD44*) biomarkers and *CD274*. **b**,** c** Gene set enrichment analysis (GSEA) of the *NECTIN4* high and low groups. **b** Enrichment plot of HALLMARK_OXIDATIVE_PHOSPHORYLATION in the *NECTIN4*-high group in patients with UTUC. **c** Enrichment plot of EPITHELIAL_MESENCHYMAL_TRANSITION in the *NECTIN4*-low group

In the Hiroshima cohort, Nectin-4 expression was associated with UPK3 and GATA3 expression and inversely associated with PD-L1 and CK 5/6 in UTUC. To further confirm these relationships, we analyzed the associations between *NECTIN4* and luminal (*GATA3, UPK3A, UPK3B, FOXA1, and ERBB2*), basal (*KRT5, KRT6A, KRT6B, KRT6C, KRT14*, and *CD44*) biomarkers and between *NECTIN4* and *CD274* (PD-L1) using the RNA-seq dataset from Fujii et al.'s UTUC study. Consistent with the findings in the Hiroshima cohort, Nectin-4 was positively associated with luminal markers and inversely associated with basal markers and *CD274* gene expression (Fig. 3A).

### Bioinformatics analysis of NECTIN4 in UTUC using the RNA-seq dataset

The 158 UTUC samples from Fujii et al. [27] were classified into *NECTIN4*-high and *NECTIN4*-low groups using an 84% cutoff. GSEA revealed that high *NECTIN4* expression was significantly associated with oxidative phosphorylation (OXPHOS), whereas low expression was linked to epithelial‒mesenchymal transition (EMT) (Fig. 3B, C).

**Fig. 4:**
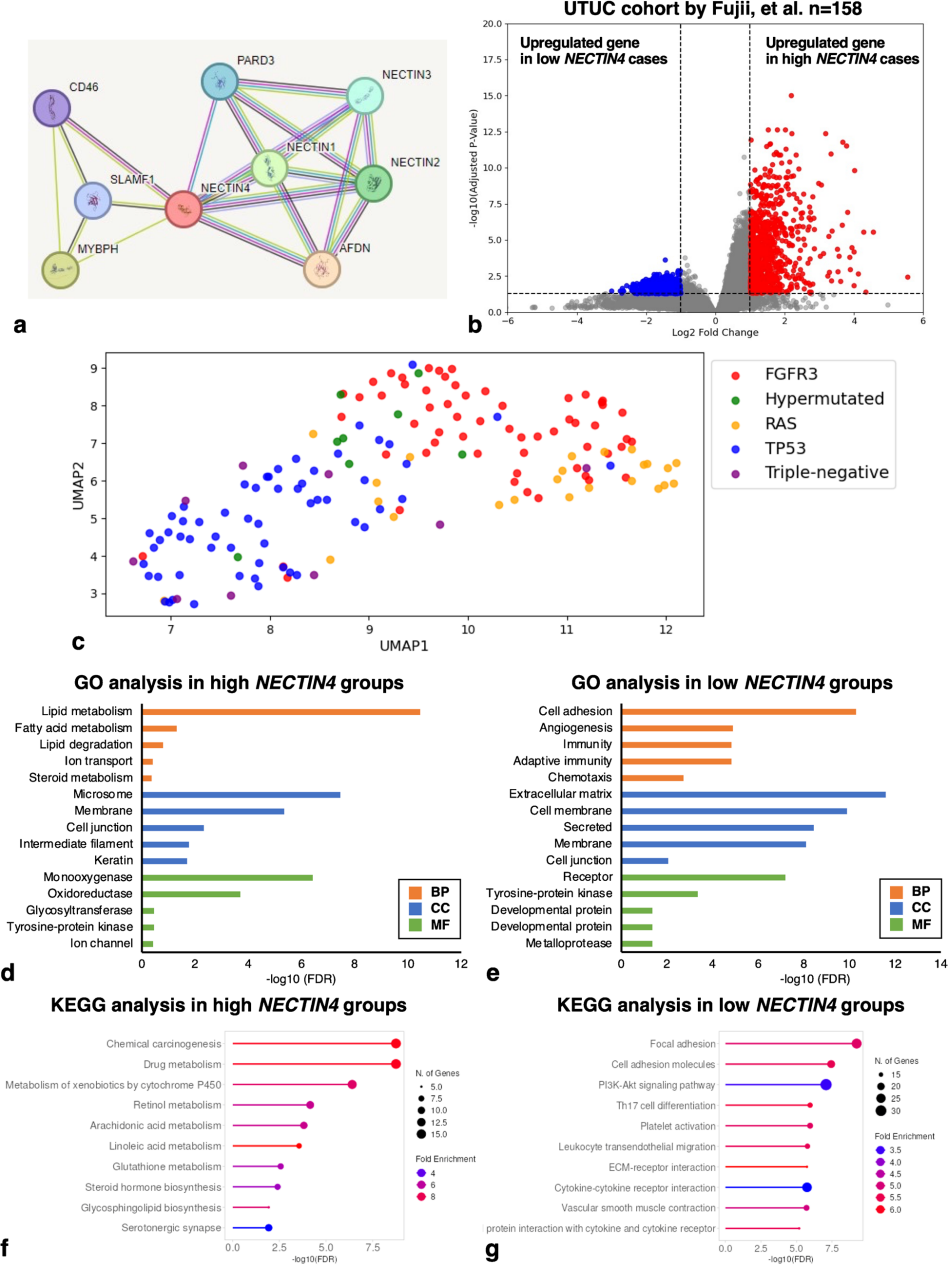
Network analysis and functional enrichment analysis of differentially expressed genes (DEGs) between high and low *NECTIN4* expression in upper tract urothelial carcinoma (UTUC). **a** Most *NECTIN4*-related proteins according to the STRING database. **b** Volcano plot of DEGs. Each point represents an individual gene. The X-axis shows the log2-fold change in gene expression (*NECTIN4*-high/low), and the Y-axis shows the -log10 adjusted *P* value. The vertical dotted lines indicate a twofold change threshold, and the horizontal dotted line denotes an adjusted *P* value of 0.05. Genes in red are upregulated in *NECTIN4*-high UTUC, whereas those in blue are downregulated. (**c**) Uniform manifold approximation and projection (UMAP) distribution plots of UTUC cases based on DEGs. **d**,** e** Gene Ontology (GO) enrichment analysis of upregulated genes in the (**d**) *NECTIN4*-high and (**e**) -low groups. **f**,** g** Kyoto Encyclopedia of Genes and Genomes (KEGG) enrichment analysis of upregulated genes in the (**f**) *NECTIN4*-high and (**g**) -low groups

PPI network analysis using STRING identified *NECTIN1, NECTIN2, NECTIN3, AFDN, PARD3, CD46, SLAMF1,* and *MYBPH* as interacting proteins (Fig. 4A). Differential expression analysis revealed 960 and 697 upregulated genes in the *NECTIN4*-high and *NECTIN4*-low groups, respectively (fold change ≥ 2, adjusted p < 0.05) (Fig. 4B). UMAP visualization of the 1,657 DEGs revealed a distinction between the FGFR3 and TP53 mutational subtypes, suggesting that *NECTIN4*-related DEGs contribute to subtype classification (Fig. 4C).

GO analysis revealed that the upregulated genes in the *NECTIN4*-high group were enriched in lipid metabolism, microsomes, membranes, monooxygenases, and oxidoreductases, whereas those in the *NECTIN4*-low group were associated with cell adhesion, the ECM, secretion, and receptors (Fig. 4D, E). KEGG analysis further revealed that *NECTIN4*-high status was associated with chemical carcinogenesis, drug metabolism, xenobiotic metabolism via cytochrome P450, and retinol metabolism. In contrast, the *NECTIN4*-low group showed enrichment in focal adhesion, cell adhesion molecules, PI3K‒Akt pathway, Th17 differentiation, and platelet activation (Fig. 4F, G).

### Investigation of Nectin-4 expression in BLCA

**Fig. 5:**
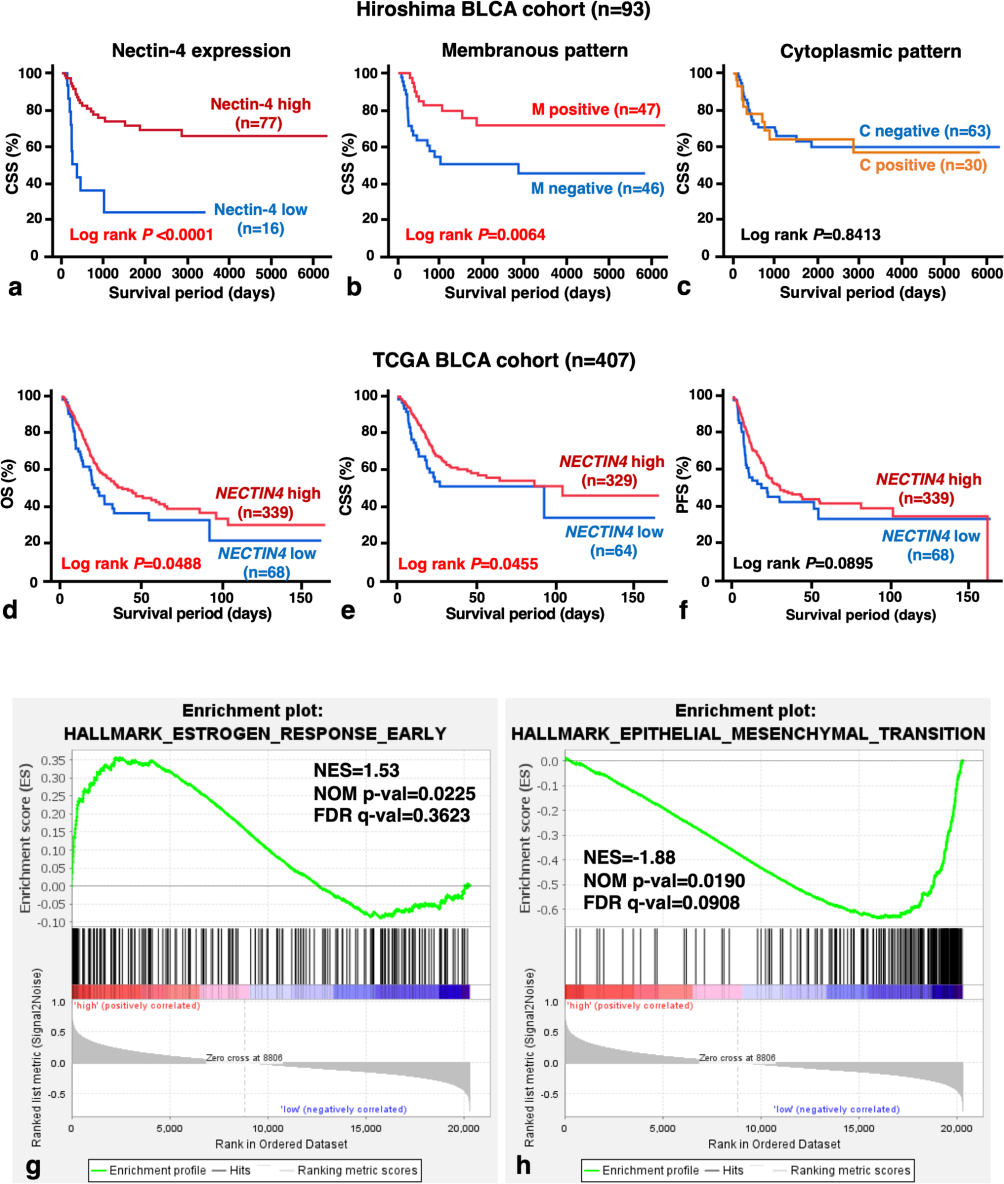
Nectin-4 expression analysis in bladder urothelial carcinoma (BLCA). **a**-**c** Kaplan‒Meier plots illustrating the cancer-specific survival (CSS) of BLCA patients stratified by overall Nectin-4 expression (**a**) overall Nectin-4 expression (high vs. low), (**b**) presence or absence of predominant membranous (M) expression, (**c**) presence or absence of predominant cytoplasmic (C) expression. (**d**-**f**) Kaplan‒Meier plot for (**d**) overall survival (OS), (**e**) CSS, and (**f**) progression-free survival (PFS) using a cutoff value of 84% for *NECTIN4* expression in the TCGA BLCA cohort. **g**,** h** Gene set enrichment analysis (GSEA) of the *NECTIN4*-high and -low groups in BLCA. **g** Enrichment plot of HALLMARK_ESTROGEN_RESPONSE_EARLY in the *NECTIN4*-high group. **h** Enrichment plot of EPITHELIAL_MESENCHYMAL_TRANSITION in the *NECTIN4*-low group

We investigated Nectin-4 expression in BLCA. IHC of 93 BLCA patients treated with RC revealed that Nectin-4 was expressed in 77 (83%) cases. Nectin-4 expression was significantly associated with younger age (*p* = 0.0052), papillary morphology (*p* = 0.0039), low tumor grade (*p* = 0.0312), and early pathological T stage (*p* = 0.0008) (Table 4), and significantly correlated with improved CSS (*p* < 0.0001, Fig. 5A). M-positive cases were significantly associated with papillary morphology (*p* = 0.0006), low tumor grade (*p* = 0.0212), and early pathological T stage (*p* = 0.0009), whereas C-positive cases showed no significant associations (Table S4). C positivity was more frequently observed in advanced BLCA (Fig. S5A). M-positivity was also significantly correlated with improved prognosis (*p* = 0.0064, Fig. 5B), while C-positivity was not associated with clinical outcomes (Fig. 5C). Additionally, survival data was stratified into stage 0/1/2 and stage 3/4 due to the relatively small sample sizes of stage 0 and 1 cases (Fig. S5B, C). In advanced-stage BLCA, Nectin-4 positivity was significantly associated with favorable prognosis (*p* = 0.0270, Fig. S5C).

**Table 4:** Relationship between Nectin-4 expression and clinicopathological parameters in bladder urothelial carcinoma

Factors in Hiroshima BLCA cohort (*n = *93)	Nectin-4 high	Nectin-4 low	*P* value
	(*n = *77)	(*n = *16)	
Age			
< 73 years (*n = *47)	44 (94%)	3 (6%)	**0.0052**
≥ 73 years (*n = *46)	33 (72%)	13 (28%)	
Sex			
Female (*n = *25)	20 (80%)	5 (20%)	0.6649
Man (*n = *68)	57 (84%)	11 (16%)	
Morphology			
Papillary (*n = *28)	28 (100%)	0 (0%)	**0.0039**
Nodular/Flat (*n = *65)	49 (75%)	16 (25%)	
Histological grade			
Low grade (*n = *40)	37 (93%)	3 (8%)	**0.0312**
High grade (*n = *53)	40 (75%)	13 (25%)	
Pathological T stage			
Stage 0/1/2 (*n = *47)	45 (96%)	2 (4%)	**0.0008**
Stage 3/4 (*n = *46)	32 (70%)	14 (30%)	
Factors in TCGA BLCA cohort (*n = *407)	*NECTIN4* high	*NECTIN4* low	*P* value
	(*n = *339)	(*n = *68)	
Age			
< 73 years (*n = *247)	203 (82%)	44 (18%)	0.4983
≥ 73 years (*n = *160)	136 (85%)	24 (15%)	
Sex			
Female (*n = *107)	80 (75%)	27 (25%)	**0.0097**
Man (*n = *300)	259 (86%)	41 (14%)	
Morphology			
Papillary (*n = *132)	122 (92%)	10 (0%)	**0.0005**
Nodular/Flat (*n = *269)	212 (79%)	57 (21%)	
Histological grade			
Low grade (*n = *21)	21 (100%)	0	**0.0327**
High grade (*n = *383)	315 (82%)	68 (18%)	
Pathological T stage			
Stage 0/1/2 (*n = *131)	104 (79%)	27 (21%)	0.1582
Stage 3/4 (*n = *274)	233 (85%)	41 (15%)	
FGFR3 mutation			
Presence(*n = *54)	53 (98%)	1 (2%)	**0.0006**
Absence (*n = *353)	286 (81%)	67 (19%)	

*P* values were calculated with Fisher’s exact test

Bold values show the statistical significance at the *P* < 0.05 level

In silico analysis [28] revealed that the cutoff value of 83% aligned with the Hiroshima cohort. *NECTIN4* expression was significantly associated with male sex (*p* = 0.0097), papillary morphology (*p* = 0.0005), low tumor grade (*p* = 0.0327), and FGFR3 mutation (*p* = 0.0006) (Table 4). *NECTIN4* expression was also significantly associated with improved overall survival (*p* = 0.0488, Fig. 5D) and CSS (*p* = 0.0455, Fig. 5E) and was marginally associated with increased PFS (*p* = 0.0895, Fig. 5F). Consistent with findings from the in silico UTUC dataset, high *NECTIN4* expression was significantly associated with improved OS (*p* = 0.0007) and CSS (*p* = 0.0019) in early-stage BLCA (Fig. S6A), but not in advanced-stage disease (Fig. S6B).

GSEA revealed that high *NECTIN4* expression was significantly associated with the estrogen response (early), whereas low expression was significantly associated with EMT (Fig. 5G, H). A volcano plot revealed 830 and 1,101 upregulated genes in the *NECTIN4*-high and *NECTIN4*-low groups, respectively (Fig. S7A). UMAP visualization of the 1,931 DEGs revealed a tendency to distinguish FGFR3 mutations from other subtypes through dimensionality reduction (Fig. S7B). GO and KEGG analyses of DEGs revealed similar results to those obtained with the UTUC dataset (Fig. S7C-F).

Since the *NECTIN4*-low group was associated with EMT in both the UTUC and BLCA cohorts, we conducted a functional analysis of EMT in vitro. We generated UMUC3 cells with forced Nectin-4 expression. Nectin-4 overexpression did not significantly change EMT marker expression (Fig. S8).

## Discussion

Previous studies have shown that enfortumab vedotin (EV), which targets Nectin-4, is effective in advanced and metastatic UC, highlighting Nectin-4 as a key molecule in tumor progression18-20]. However, its clinicopathological role remains controversial. In our study, Nectin-4 was more frequently overexpressed in tumors than in normal urothelium and was associated with papillary morphology, low grade, early pT stage, and a favorable prognosis in both UTUC and BLCA. Similar trends were confirmed in public datasets using the same cutoff value as the Hiroshima cohort. When subgroup analyses were conducted separately for early- and advanced-stage disease, a trend toward improved prognosis was observed in advanced-stage cases within the Hiroshima cohort; however, some of the results did not reach statistical significance, possibly due to the limited sample size. Interestingly, high *NECTIN4* expression was significantly associated with favorable prognosis in early-stage disease in both the in silico UTUC and BLCA datasets. These findings may offer new insights into the prognostic relevance of Nectin-4 expression in urothelial carcinoma.

In contrast, Tomiyama et al. reported no prognostic association or even suggested that high Nectin-4 expression was a poor prognostic factor [23]. This discrepancy may stem from differences in cutoff values and patient backgrounds. For example, in our analysis of the public database using the median cutoff, no prognostic difference was observed. However, when 84% of cases were defined as Nectin-4-high (consistent with our cohort), there was a significant prognostic difference. A similar trend was observed in BLCA. Additionally, Tomiyama et al.'s cohort included a high proportion of advanced-stage UTUC (T2/3/4, *n = *62/99) and high-grade (*n = *84/99) cases, whereas ours included a substantial number of early-stage and low-grade cases [23]. Selection bias may have influenced the results. Notably, Tomiyama et al. conducted their analysis using tissue microarrays, which could be impacted by tumor heterogeneity. Supporting our findings, Garczyk et al. reported Nectin-4 positivity in 91% of nonmuscle-invasive BLCA cases, with reduced expression in stromal invasive regions relative to noninvasive areas of Ta/T1 high-grade BLCA [24]. Moreover, a recent study demonstrated that Nectin-4 expression rates were significantly higher in patients with lower stages and luminal subtypes than in those with the basal or mesenchymal molecular subtypes [26]. Another study revealed an association between Nectin-4 expression and luminal subtypes in BLCA [25]. The luminal subtype generally has a favorable prognosis. While further research is needed, these results suggest that Nectin-4 is highly expressed in early-stage UC and may influence the prognosis depending on its expression pattern and localization.

In addition to assessing Nectin-4 expression, we examined its subcellular localization, an aspect rarely addressed in previous reports. Membranous Nectin-4 expression was associated with papillary morphology, low tumor grade, and early pathological stage in both UTUC and BLCA, and correlated with favorable prognosis in BLCA. Interestingly, cytoplasmic localization was more frequent in advanced-stage tumors compared to early-stage tumors, suggesting that predominant membranous Nectin-4 expression may reflect more favorable clinicopathological characteristics. In addition, Nectin-4 is currently gaining attention as a therapeutic target, especially in the context of ADCs [17]. The dependency of EV efficacy on membranous Nectin-4 expression levels, as reported in recent studies [21, 22], highlights the potential clinical utility of evaluating not only the expression level but also the localization pattern of Nectin-4 in UC.

In terms of molecular subtypes, prior studies reported high Nectin-4 expression in luminal subtypes and low expression in basal subtypes [25, 26]. Our IHC results support this, with positive correlations between Nectin-4 and luminal markers (UPK3 and GATA3) and an inverse relationship with the basal marker CK5/6. These patterns were also validated in Fujii et al.’s UTUC dataset. Moreover, in our UTUC cohort, Nectin-4 expression was marginally associated with FGFR3 expression, echoing findings from both Fujii et al. and the TCGA MIBC cohort, where *NECTIN4* was correlated with *FGFR3* mutation. FGFR3 is generally associated with a better prognosis, lower grade, and earlier stage [30, 31]. Given the high frequency of *FGFR3* mutations in luminal papillary UC, it is plausible that *NECTIN4* and *FGFR3* are closely related.

These findings suggest that high Nectin-4 expression is primarily localized to the cell membrane in well-differentiated UC, whereas cytoplasmic staining or loss of expression may increase in poorly differentiated tumors. Indeed, the *NECTIN4*-low group was significantly associated with EMT, which was consistent with the lower Nectin-4 expression observed in the basal subtype. Given that EV efficacy likely depends on membranous Nectin-4, as with HER2-targeted antibodies and HER2-ADCs, assessing Nectin-4 localization may enhance therapeutic evaluation. This might explain why the clinical efficacy of EV plus pembrolizumab (EV + Pem) is notably greater than that of EV alone, as the first-line setting often includes well-differentiated tumors with higher membranous Nectin-4 expression. In general, the basal subtype is known to be associated with drug resistance [26, 32], indicating that basal-like tumors may predominate following chemotherapy. When EV is used for second- or third-line treatment, its efficacy may be reduced, as Nectin-4 is primarily expressed in luminal types. Indeed, a recent study revealed that patients with low Nectin-4 expression benefited more from platinum-based chemotherapy in both adjuvant and neoadjuvant settings [26].

Notably, “Nectin-4 et al. IS”, which incorporates Nectin-4, UPK3, GATA3, and PD-L1, showed a significant association with the prognosis in UTUC and served as an independent predictor for PFS. This index showed high predictive accuracy for patient outcomes. Additionally, machine learning models such as the SVM and random forest classifiers, which incorporate “Nectin-4 et al. IS” alongside clinicopathological factors, predict a poor prognosis with greater accuracy than clinicopathological factors alone. These findings suggest that combining Nectin-4 with subtype markers and PD-L1 enhances prognostic precision and may guide tailored therapies.

Bioinformatics analysis revealed that *NECTIN4* is enriched in various functions and pathways, including OXPHOS. Additionally, the STRING analysis indicated that *NECTIN4* interacts with molecules such as *PARD3, SLAMF4,* and *CD46*, suggesting its significant role in cell adhesion and the tumor microenvironment. PARD3 and CD46 have been reported in BLCA, and both play crucial roles in BLCA tumorigenesis [33, 34]. Notably, high CD46 and PARD3 expression is a favorable prognostic factor in BLCA [34, 35]. GO and KEGG pathway analyses revealed that genes in the *NECTIN4*-high group were associated with chemical carcinogenesis, drug metabolism and xenobiotic metabolism, aligning with the link between UC and environmental carcinogens [36]. Conversely, the *NECTIN4*-low group was associated with EMT, cell adhesion, focal adhesion and ECM. Focal adhesion is also implicated in PI3K-AKT pathway activation, which is central to malignant behaviors, including chemotherapy resistance, in various cancers, including UC [37, 38]. Overall, these findings underscore the prognostic significance of the Nectin-4 status, as it influences critical biological processes driving malignant behaviors in UC. These findings highlight the multifaceted role of Nectin-4 in promoting UC tumorigenesis and emphasize its potential as both a prognostic marker and therapeutic target.

This study has several limitations. Due to its retrospective design, prospective validation is required. While we assessed Nectin-4 expression and localization, the impact of membrane vs. cytoplasmic staining on treatment efficacy remains unclear and requires clinical evaluation. Intratumoral heterogeneity in Nectin-4 protein expression was observed in some cases; thus, further analyses are required to better understand its variability. Despite our findings linking Nectin-4 to better clinicopathological features, it has also been reported to be a poor prognostic factor in various carcinomas, including UTUC. In addition, while stratified survival analyses were conducted, the number of events in early-stage cases within our UTUC and BLCA cohorts was limited. Further large-scale studies are warranted to clarify the prognostic significance of Nectin-4 expression in urothelial carcinoma. Moreover, although changes in EMT were not evident in Nectin-4-overexpressing cells, the result was limited to one cell line; thus, broader studies are warranted to clarify the role of Nectin-4 in UC biology.

## Conclusions

This study comprehensively characterized Nectin-4 expression in both upper and lower tract UC. Notably, Nectin-4 expression was associated with better clinicopathological features, luminal subtype markers and *FGFR3* mutation. Assessing Nectin-4 expression and localization patterns may provide critical insights for precision medicine and could contribute to identifying patient subgroups with potentially better outcomes.

## Supplementary Information

Below is the link to the electronic supplementary material.

Supplementary file1 (TIFF 44308 KB)

Supplementary file2 (TIFF 45274 KB)

Supplementary file3 (TIFF 42315 KB)

Supplementary file4 (TIFF 12849 KB)

Supplementary file5 (TIFF 35084 KB)

Supplementary file6 (TIFF 20086 KB)

Supplementary file7 (TIFF 45470 KB)

Supplementary file8 (TIFF 4037 KB)

Supplementary file9 (DOCX 51 KB)

Supplementary file10 (DOCX 15 KB)

Supplementary file11 (DOCX 47 KB)

## Data Availability

All research data and material will be made available upon request. Most data are included in the main manuscript.
