# Supplementary figures and images for: Nectin-4 expression in upper and lower tract urothelial carcinoma: correlation with early-stage disease and prognostic relevance
Source: Virchows Arch. 2025 Jun 27;488(6):1227–42. doi: 10.1007/s00428-025-04164-9 (PMC13264583; doi:10.1007/s00428-025-04164-9)

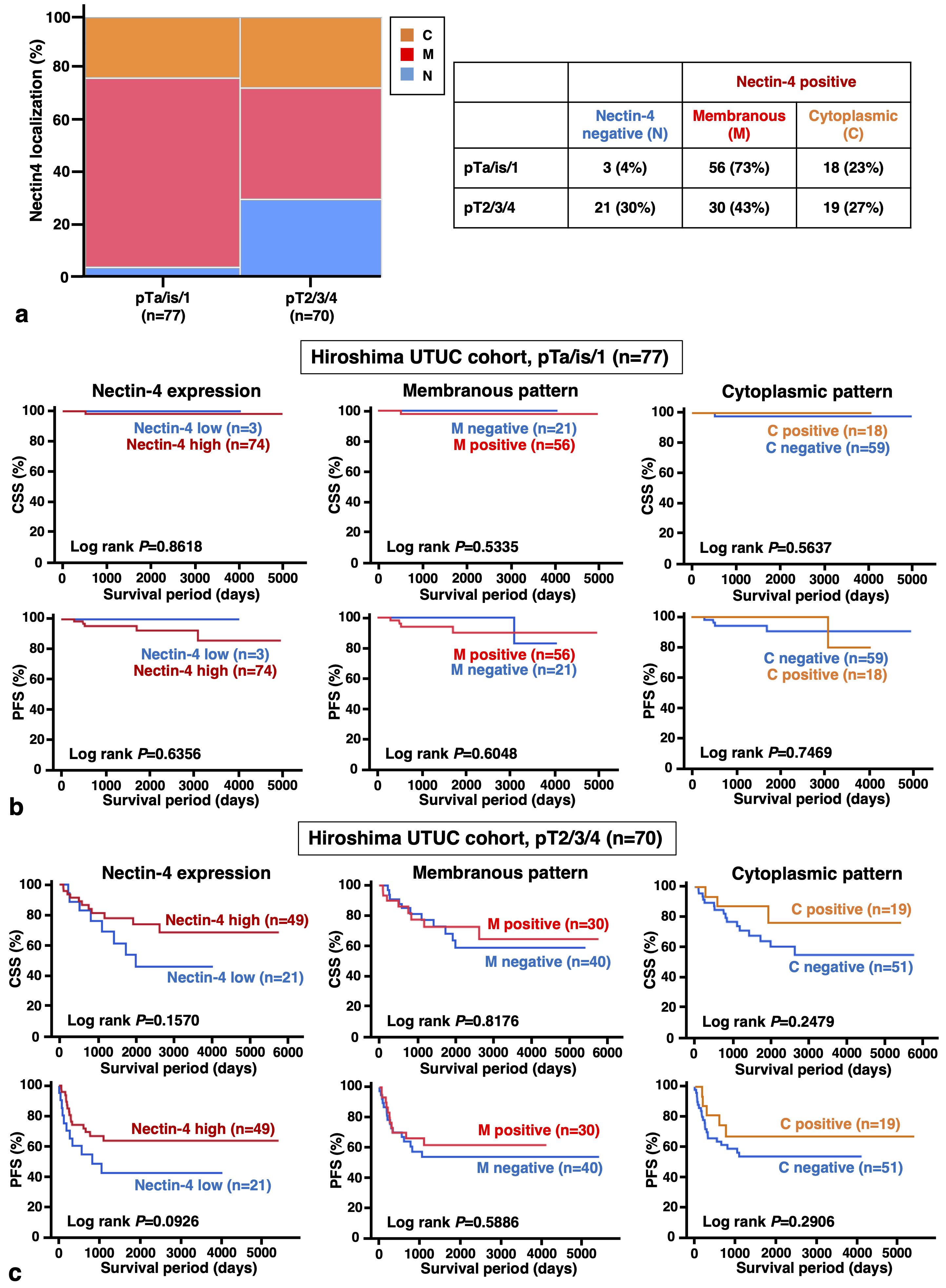

Supplement: Supplementary file 1 — Supplementary file1 (TIFF 44308 KB) [file 428_2025_4164_MOESM1_ESM.tiff]

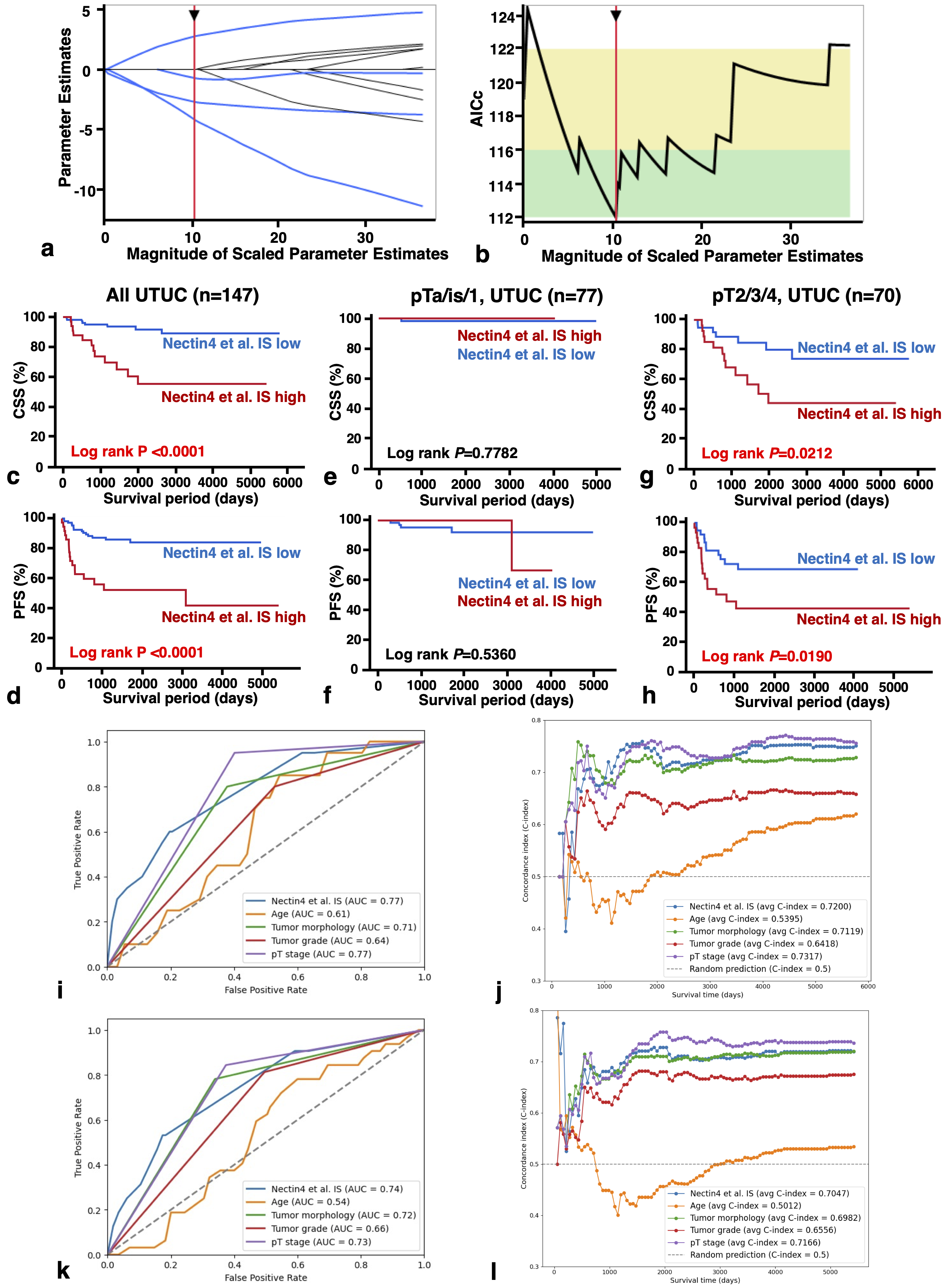

Supplement: Supplementary file 2 — Supplementary file2 (TIFF 45274 KB) [file 428_2025_4164_MOESM2_ESM.tiff]

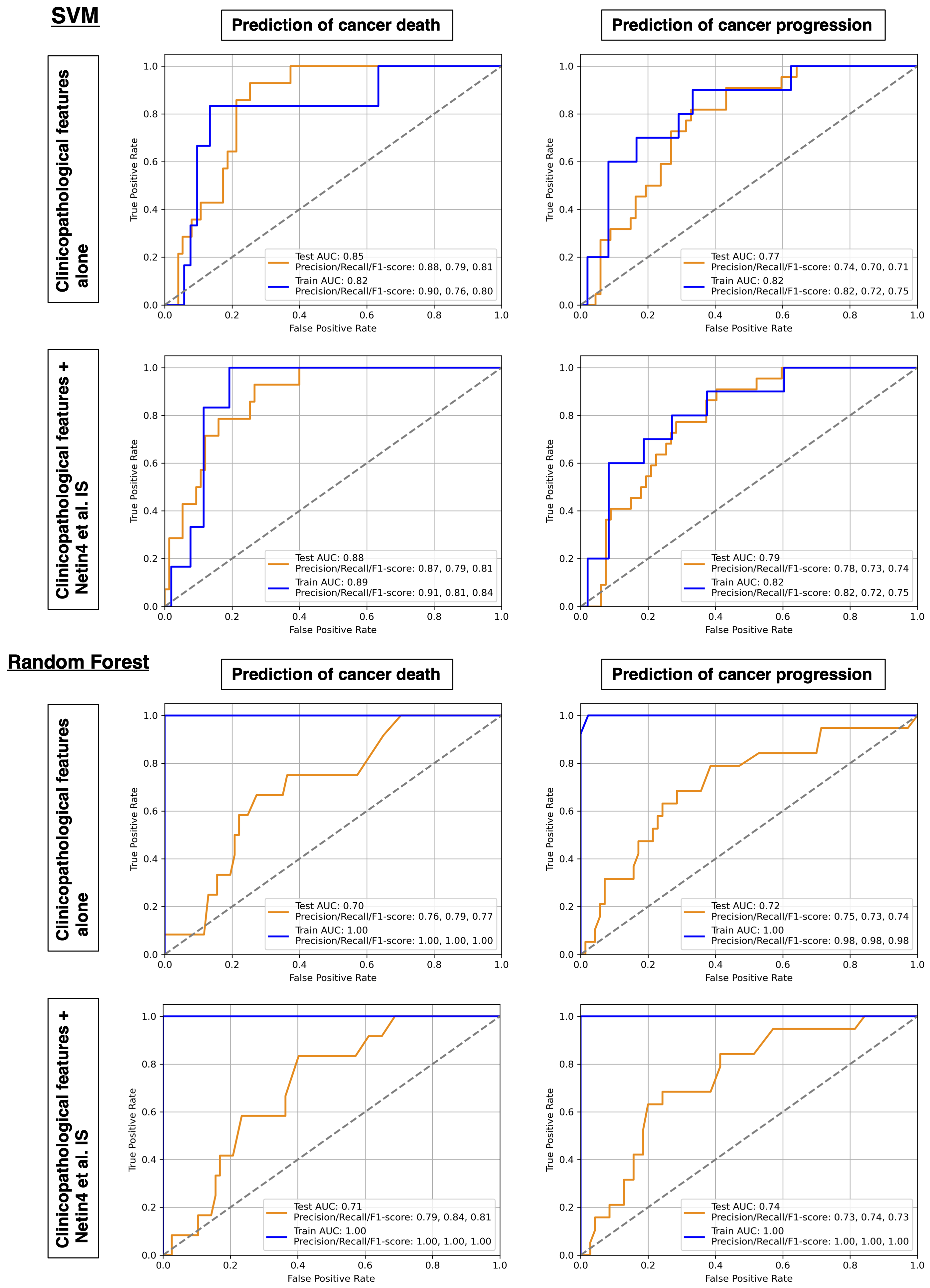

Supplement: Supplementary file 3 — Supplementary file3 (TIFF 42315 KB) [file 428_2025_4164_MOESM3_ESM.tiff]

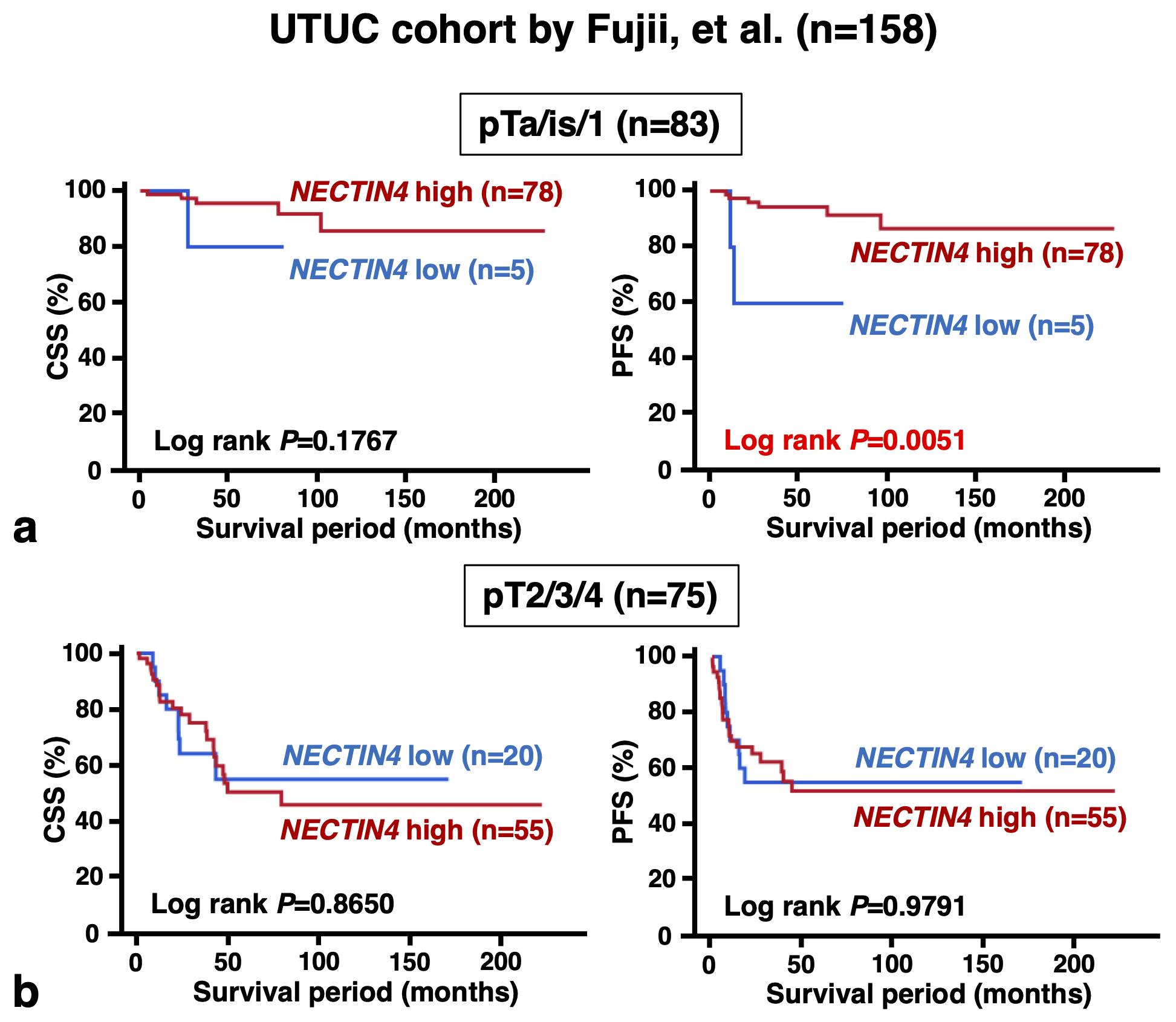

Supplement: Supplementary file 4 — Supplementary file4 (TIFF 12849 KB) [file 428_2025_4164_MOESM4_ESM.tiff]

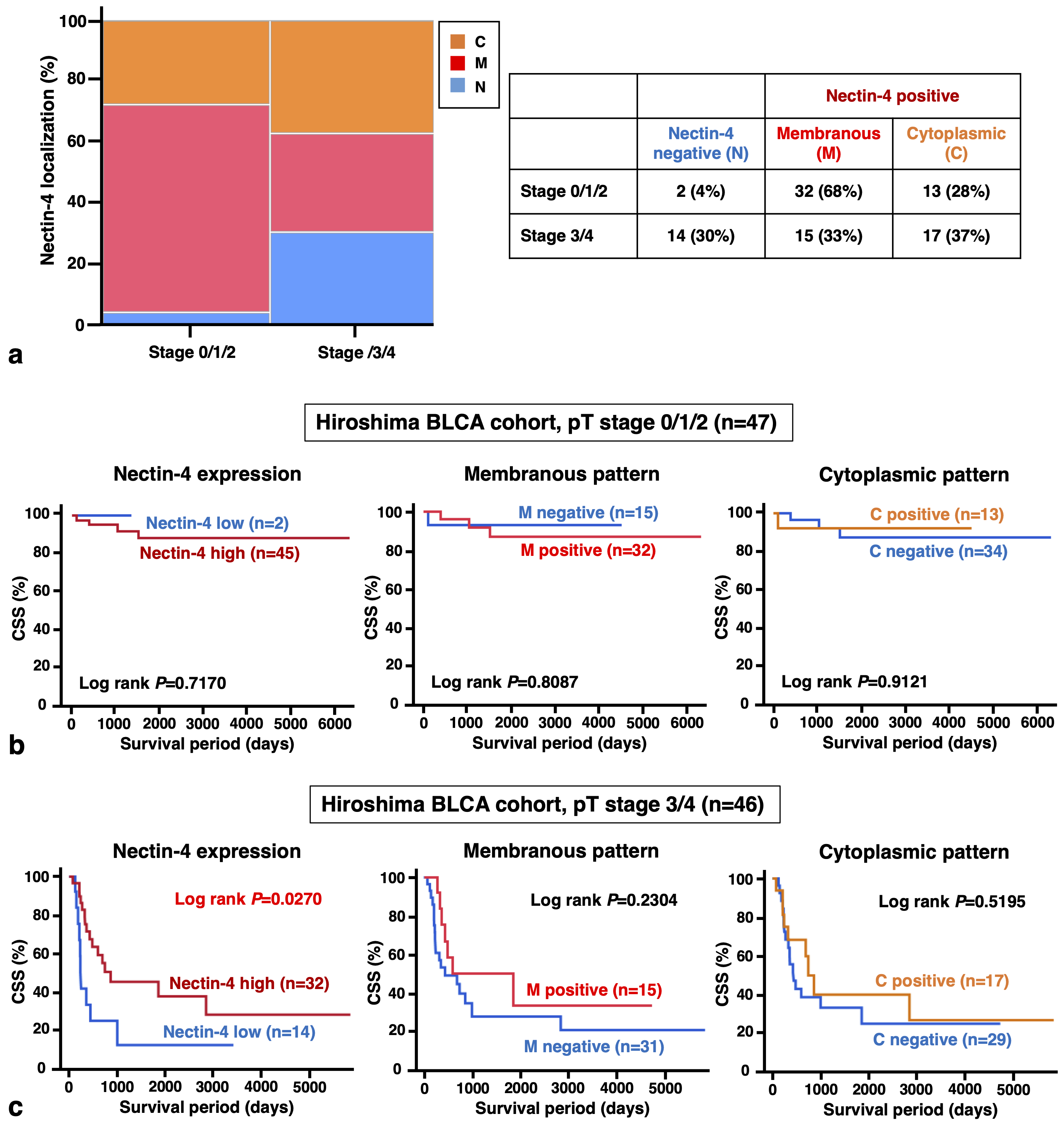

Supplement: Supplementary file 5 — Supplementary file5 (TIFF 35084 KB) [file 428_2025_4164_MOESM5_ESM.tiff]

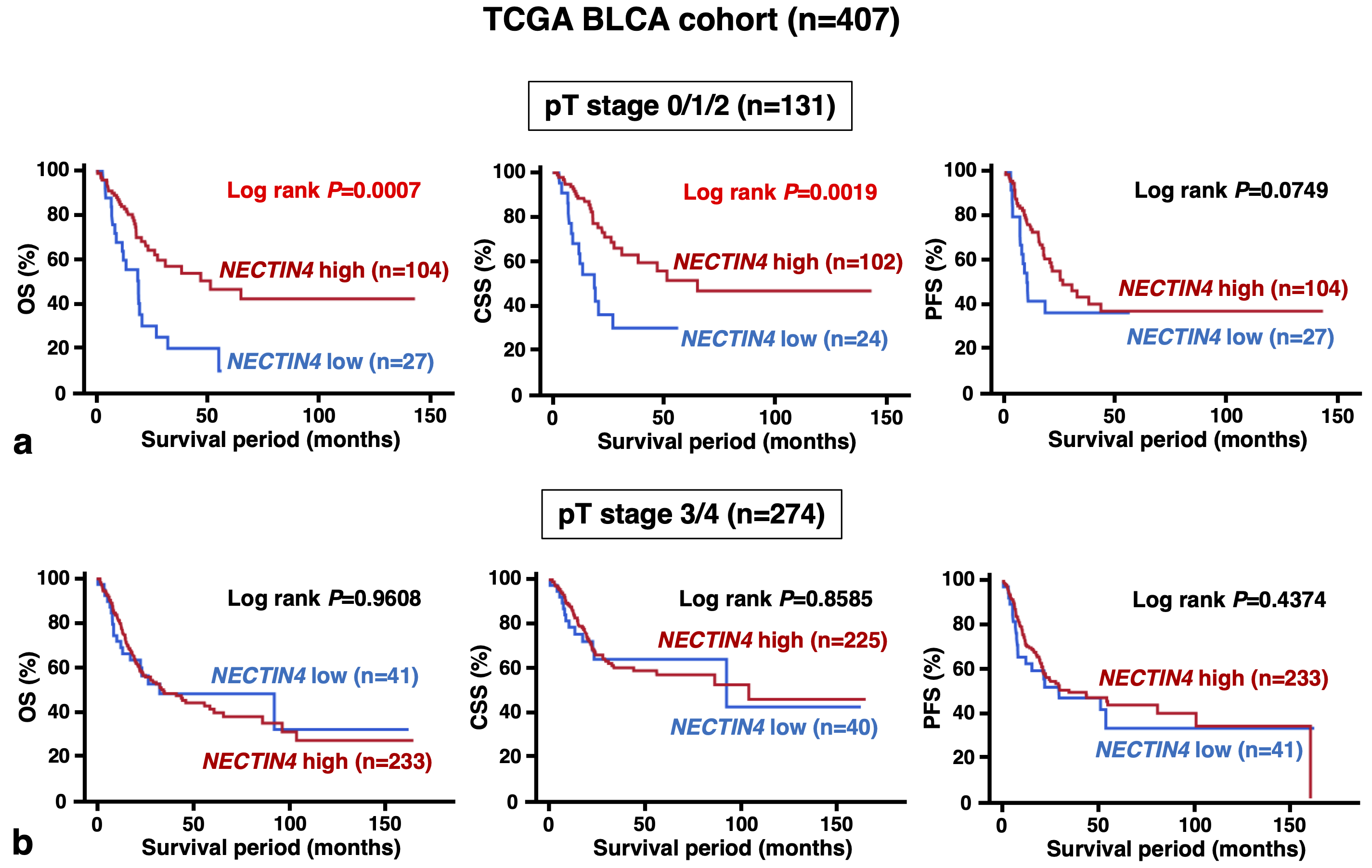

Supplement: Supplementary file 6 — Supplementary file6 (TIFF 20086 KB) [file 428_2025_4164_MOESM6_ESM.tiff]

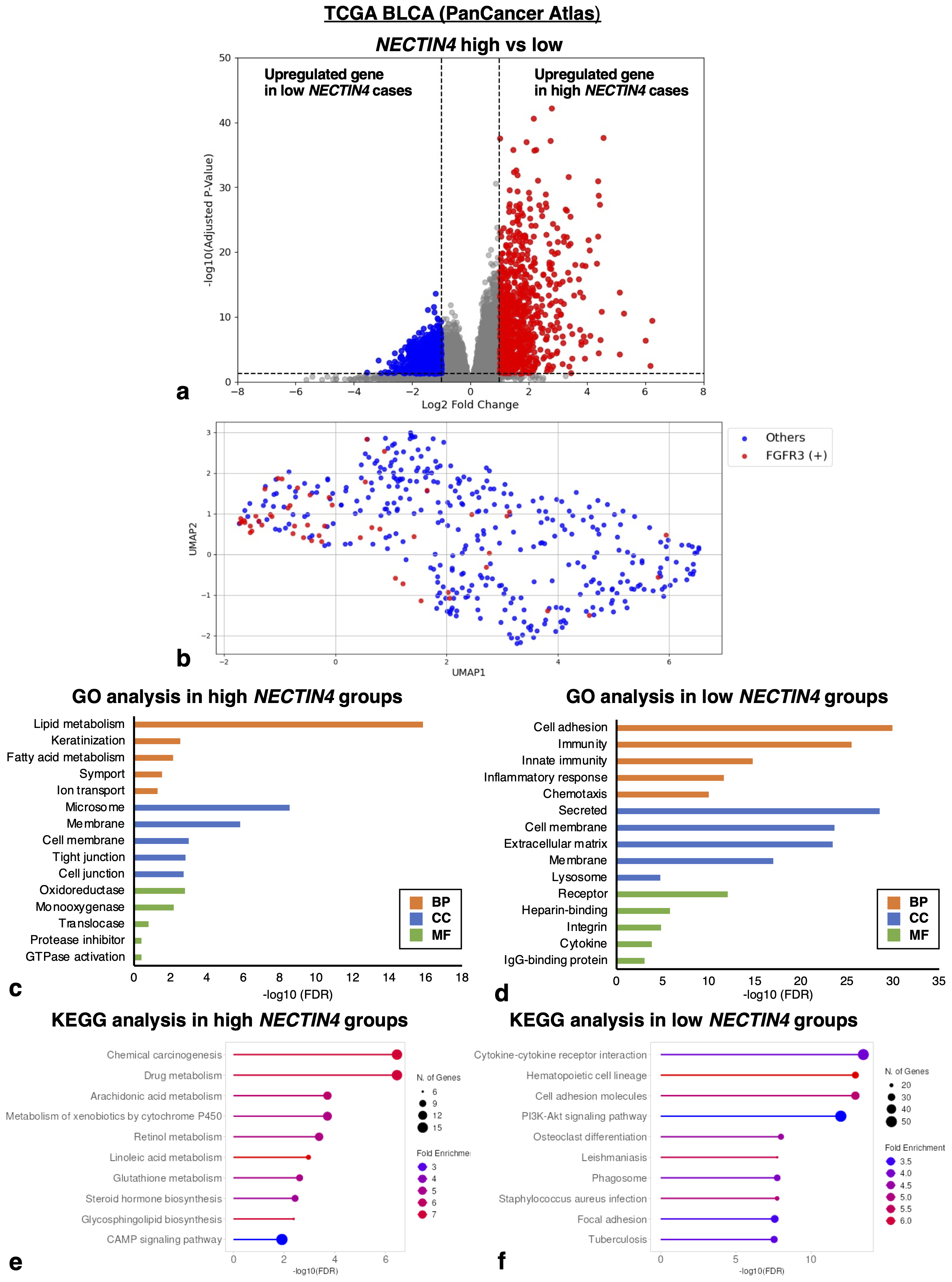

Supplement: Supplementary file 7 — Supplementary file7 (TIFF 45470 KB) [file 428_2025_4164_MOESM7_ESM.tiff]

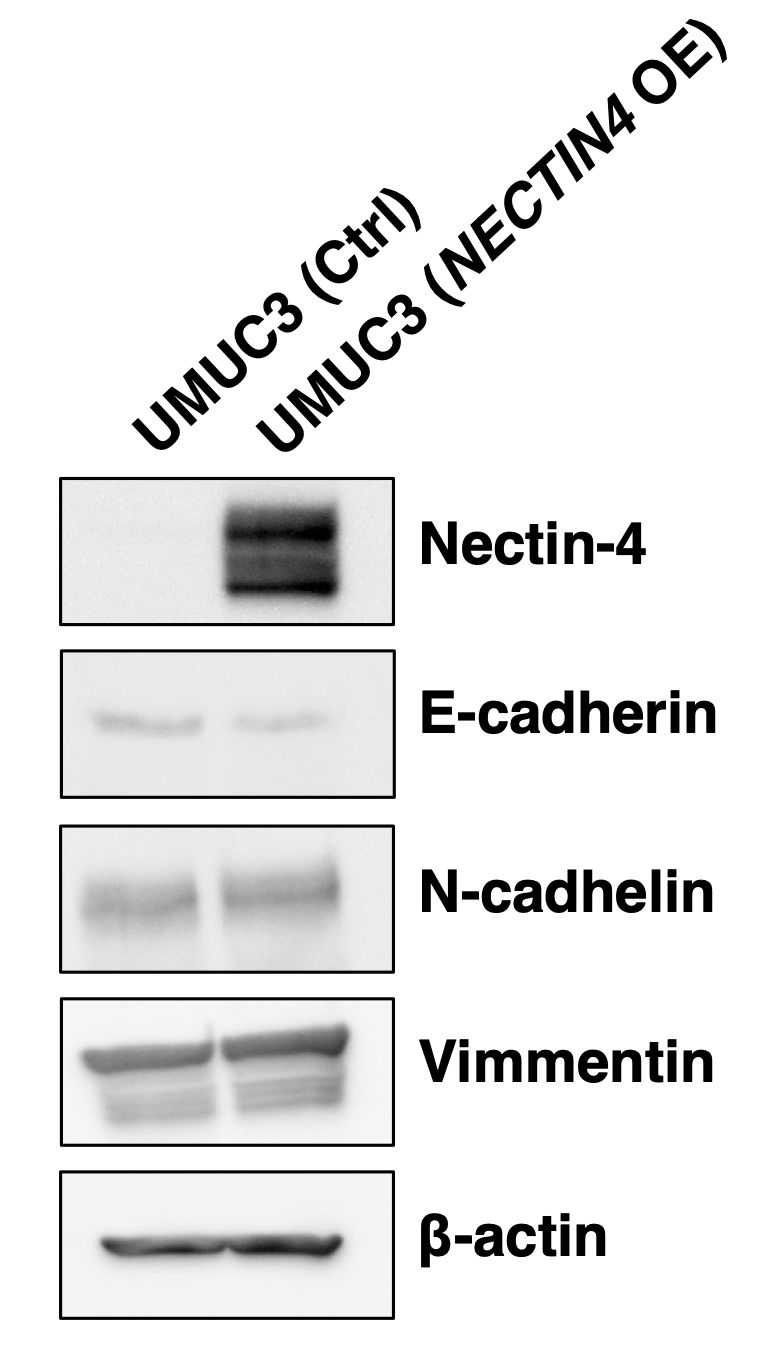

Supplement: Supplementary file 8 — Supplementary file8 (TIFF 4037 KB) [file 428_2025_4164_MOESM8_ESM.tiff]
