# Supplementary material for: Nectin-4 expression in upper and lower tract urothelial carcinoma: correlation with early-stage disease and prognostic relevance
Source: Virchows Arch. 2025 Jun 27;488(6):1227–42. doi: 10.1007/s00428-025-04164-9 (PMC13264583; doi:10.1007/s00428-025-04164-9)
Supplement: Supplementary file 9 — Supplementary file9 (DOCX 51 KB) [file 428_2025_4164_MOESM9_ESM.docx]

**Table S1. Information of antibodies in western blot analysis**

| Antibody | Host / Isotype | Product code | Company |
| --- | --- | --- | --- |
| Nectin4 monoclonal antibody | Rabbit / IgG | ab192033 | Abcam |
| Beta Actin monoclonal antibody | Mouse / IgG2b | 66009-1-Ig | Proteintech |
| E-cadherin Polyclonal antibody | Rabbit / IgG | 20874-1-AP | Proteintech |
| N-Cadherin monoclonal antibody | Rabbit / IgG | #13116 | Cell Signaling |
| Vimentin monoclonal antibody | Rabbit / IgG | #5741 | Cell Signaling |

**Table S2. Relationship between membranous Nectin-4 expression and clinicopathological parameters in 147 cases of upper tract urothelial carcinoma.**

|  | Membranous Nectin-4 expression | | *P* value |
| --- | --- | --- | --- |
|  | Positive (%) | Negative (%) |  |
| Age |  |  |  |
| <73 years (n=74) | 46 (63%) | 28 (38%) | 0.4049 |
| ≥73 years (n=73) | 40 (55%) | 33 (45%) |  |
| Sex |  |  |  |
| Female (n=33) | 18 (55%) | 15 (45%) | 0.6892 |
| Man (n=114) | 68 (60%) | 46 (40%) |  |
| Lateralization |  |  |  |
| Right (n=66) | 40 (61%) | 26 (39%) | 0.7368 |
| Left (n=81) | 46 (57%) | 35 (43%) |  |
| Location |  |  |  |
| Renal pelvis (n=69) | 35 (51%) | 34 (49%) | 0.0935 |
| Ureter (n=78) | 51 (65%) | 27 (35%) |  |
| Morphology |  |  |  |
| **Papillary (n=83)** | 58 (70%) | 25 (30%) | **0.0014** |
| Nodular/Flat (n=64) | 28 (44%) | 36 (56%) |  |
| Histological grade |  |  |  |
| **Low grade (n=64)** | 44 (69%) | 20 (31%) | **0.0295** |
| High grade (n=83) | 42 (51%) | 41 (49%) |  |
| Pathological T stage |  |  |  |
| **pTa/is/1 (n=77)** | 56 (73%) | 21 (27%) | **0.0004** |
| pT2/3/4 (n=70) | 30 (43%) | 40 (57%) |  |
| *P* values were calculated with Fisher’s exact test.  Bold values show the statistical significance at the *P* < 0.05 level. | | | |

**Table S3. Relationship between cytoplasmic Nectin-4 expression and clinicopathological parameters in 147 cases of upper tract urothelial carcinoma.**

|  | Cytoplasmic Nectin-4 expression | | *P* value |
| --- | --- | --- | --- |
|  | Positive (%) | Negative (%) |  |
| Age |  |  |  |
| <73 years (n=74) | 18 (24%) | 56 (76%) | 0.8509 |
| ≥73 years (n=73) | 19 (26%) | 54 (74%) |  |
| Sex |  |  |  |
| Female (n=33) | 11 (33%) | 22 (67%) | 0.2563 |
| Man (n=114) | 26 (23%) | 88 (77%) |  |
| Lateralization |  |  |  |
| Right (n=66) | 12 (18%) | 54 (82%) | 0.0882 |
| Left (n=81) | 25 (31%) | 56 (69%) |  |
| Location |  |  |  |
| **Renal pelvis (n=69)** | 25 (36%) | 44 (63%) | **0.0044** |
| Ureter (n=78) | 12 (15%) | 66 (85%) |  |
| Morphology |  |  |  |
| Papillary (n=83) | 21 (25%) | 62 (75%) | 1.0000 |
| Nodular/Flat (n=64) | 16 (25%) | 48 (75%) |  |
| Histological grade |  |  |  |
| Low grade (n=64) | 18 (28%) | 46 (72%) | 0.5659 |
| High grade (n=83) | 19 (23%) | 64 (77%) |  |
| Pathological T stage |  |  |  |
| pTa/is/1 (n=77) | 18 (23%) | 59 (77%) | 0.7041 |
| pT2/3/4 (n=70) | 19 (27%) | 51 (73%) |  |
| *P* values were calculated with Fisher’s exact test.  Bold values show the statistical significance at the *P* < 0.05 level. | | | |

**Table S4. Relationship between Nectin-4 localization patterns and clinicopathological parameters in 93 cases of bladder urothelial carcinoma.**

|  | Membranous Nectin-4 expression | | *P* value |
| --- | --- | --- | --- |
|  | Positive (%) | Negative (%) |  |
| Age |  |  |  |
| <73 years (n=47) | 27 (57%) | 20 (43%) | 0.2156 |
| ≥73 years (n=46) | 20 (43%) | 26 (57%) |  |
| Sex |  |  |  |
| Female (n=25) | 12 (48%) | 13 (52%) | 0.8180 |
| Man (n=68) | 35 (51%) | 33 (49%) |  |
| Morphology |  |  |  |
| **Papillary (n=28)** | 22 (79%) | 6 (21%) | **0.0006** |
| Nodular/Flat (n=65) | 25 (38%) | 40 (62%) |  |
| Histological grade |  |  |  |
| **Low grade (n=40)** | 26 (65%) | 14 (35%) | **0.0212** |
| High grade (n=53) | 21 (40%) | 32 (60%) |  |
| Pathological T stage |  |  |  |
| **Stage 0/1/2 (n=47)** | 32 (68%) | 15 (32%) | **0.0009** |
| Stage 3/4 (n=46) | 15 (33%) | 31 (67%) |  |
|  | Cytoplasmic Nectin-4 expression | | *P* value |
|  | Positive (%) | Negative (%) |  |
| Age |  |  |  |
| <73 years (n=47) | 17 (36%) | 30 (64%) | 0.5072 |
| ≥73 years (n=46) | 13 (28%) | 33 (72%) |  |
| Sex |  |  |  |
| Female (n=25) | 8 (32%) | 17 (68%) | 1.0000 |
| Man (n=68) | 22 (32%) | 46 (68%) |  |
| Morphology |  |  |  |
| Papillary (n=28) | 6 (21%) | 22 (79%) | 0.1572 |
| Nodular/Flat (n=65) | 24 (37%) | 41 (63%) |  |
| Histological grade |  |  |  |
| Low grade (n=40) | 11 (28%) | 29 (73%) | 0.5025 |
| High grade (n=53) | 19 (36%) | 34 (64%) |  |
| Pathological T stage |  |  |  |
| Stage 0/1/2 (n=47) | 13 (28%) | 34 (72%) | 0.3804 |
| Stage 3/4 (n=46) | 17 (37%) | 29 (63%) |  |
| *P* values were calculated with Fisher’s exact test.  Bold values show the statistical significance at the *P* < 0.05 level. | | | |
