# Supplementary material for: Nectin-4 expression in upper and lower tract urothelial carcinoma: correlation with early-stage disease and prognostic relevance
Source: Virchows Arch. 2025 Jun 27;488(6):1227–42. doi: 10.1007/s00428-025-04164-9 (PMC13264583; doi:10.1007/s00428-025-04164-9)
Supplement: Supplementary file 10 — Supplementary file10 (DOCX 15 KB) [file 428_2025_4164_MOESM10_ESM.docx]

**Supplementary text**

**Gene set enrichment analysis (GSEA)**

The GSEA software was downloaded from https://www.gsea-msigdb.org, and the samples were divided into *NECTIN4*-high and *NECTIN4*-low groups. The gene sets of “h.all.v2024.1.\\hs.symbols.gmt” were downloaded from the Molecular Signatures Database. The number of permutations was set at 1000. Normalized enrichment scores, the nominal *P* value (NOM *P* value), and false discovery rate (FDR) Q value were acquired. NOM *P*<0.05 and FDR Q<0.25 were considered to indicate significant enrichment.

**Protein‒protein interaction (PPI) network analysis**

The Search Tool for the Retrieval of Interacting Genes (STRING) (https://string-db.org/) was used to construct a protein‒protein interaction (PPI) network between *NECTIN4* and related proteins. In both databases, the species was set to "*Homo sapiens*." In STRING, the minimum required interaction score was set to high confidence values (0.700).

**Screening of differentially expressed genes (DEGs)**

According to the RNA-seq database, we divided patients into *NECTIN4*-high and *NECTIN4*-low groups, and differentially expressed genes (DEGs) were screened via a volcano plot. The threshold was a 1.5-fold change, and adjusted *P*<0.05. The volcano map of DEGs was constructed by installing Python 3.0 on Jupyter Notebook (ver. 6.3.0), an interactive computing notebook environment. The scripts are presented in Supplementary Data 1.

**Gene ontology and functional enrichment**

To determine the biological function of *NECTIN4* and its related differentially expressed genes (DEGs), we annotated them with Gene Ontology (GO), Kyoto Encyclopedia of Genes and Genomes (KEGG), DAVID v6.8 (<https://david.ncifcrf.gov/>) and Shiny Go 0.77 (<http://bioinformatics.sdstate.edu/go77/>). Gene Ontology analysis results were categorized into biological process (BP), molecular function (MF), and cellular component (CC) categories. Results with FDR<0.05 were considered statistically significant.
