# Supplementary material for: Nectin-4 expression in upper and lower tract urothelial carcinoma: correlation with early-stage disease and prognostic relevance
Source: Virchows Arch. 2025 Jun 27;488(6):1227–42. doi: 10.1007/s00428-025-04164-9 (PMC13264583; doi:10.1007/s00428-025-04164-9)
Supplement: Supplementary file 11 — Supplementary file11 (DOCX 47 KB) [file 428_2025_4164_MOESM11_ESM.docx]

1. **Python cord for volcano plot**

import pandas as pd

import numpy as np

from scipy import stats

import statsmodels.stats.multitest as smm

import matplotlib.pyplot as plt

# Load the CSV file

file_path = 'TCGA UTUC Nectin4.csv' # Path to the CSV file

data = pd.read_csv(file_path, index_col=0)

# Replace 0 with 0.1

data.replace(0, 0.1, inplace=True)

# Calculate the proportion of 0.1 values for each gene

threshold = 0.2 # Threshold: genes with ≥80% of values being 0.1

low_variation_genes = (data == 0.1).mean(axis=1) >= threshold

# Identify genes to be removed

genes_to_remove = low_variation_genes[low_variation_genes].index

print(f"Number of genes to be removed: {len(genes_to_remove)}")

# Remove genes with ≥80% of values being 0.1 across all samples

filtered_data = data.loc[~low_variation_genes]

# Debug: check the shape of the filtered dataset

print(f"Shape of filtered data: {filtered_data.shape}")

# Save the filtered dataset if needed

filtered_data.to_csv('cleaned_UTUC data part2.csv')

# Debug: reload and verify the saved data

saved_data = pd.read_csv('cleaned_UTUC data.csv', index_col=0)

print(f"Shape of saved data: {saved_data.shape}")

# Reload the cleaned data

data = pd.read_csv('cleaned_UTUC data.csv', index_col=0)

# Classify samples based on Nectin4 expression

nectin4_values = data.loc['Nectin4']

high_samples = nectin4_values[nectin4_values == 1].index # High expression group

low_samples = nectin4_values[nectin4_values == 0].index # Low expression group

# Create separate DataFrames for each group

high_data = data[high_samples]

low_data = data[low_samples]

# Drop the Nectin4 row

data = data.drop(index='Nectin4')

# Initialize results list

results = []

# Debug: check shapes and types

print(f"High group shape: {high_data.shape}, Low group shape: {low_data.shape}")

# Iterate over each gene

for gene in data.index:

high_expression = high_data.loc[gene].squeeze().replace(0, np.nan).dropna().astype(float)

low_expression = low_data.loc[gene].squeeze().replace(0, np.nan).dropna().astype(float)

if isinstance(high_expression, pd.Series) and isinstance(low_expression, pd.Series):

print(f"Gene: {gene}, High group shape: {high_expression.shape}, Low group shape: {low_expression.shape}")

print(f"Data types - High: {high_expression.dtype}, Low: {low_expression.dtype}")

if len(high_expression) > 1 and len(low_expression) > 1:

t_stat, p_value = stats.ttest_ind(high_expression, low_expression, equal_var=False)

log2_fc = np.log2(high_expression.mean() / low_expression.mean())

results.append([gene, log2_fc, p_value])

else:

print(f"Insufficient sample size for gene {gene}")

else:

print(f"Error: Expression data for gene {gene} is not in Series format.")

# Convert results to DataFrame

results_df = pd.DataFrame(results, columns=['Gene', 'Log2_FC', 'P_Value'])

# Save the results

results_df.to_csv('DEGs_results.csv', index=False)

# Adjust p-values using Benjamini-Hochberg FDR

alpha = 0.05

results_df['Adjusted_P_Value'] = smm.multipletests(results_df['P_Value'], alpha=alpha, method='fdr_bh')[1]

# Extract significant genes (adjusted p-value < 0.05)

significant_genes = results_df[results_df['Adjusted_P_Value'] < alpha]

print(f"Number of significant DEGs: {len(significant_genes)}")

# Save significant DEGs

significant_genes.to_csv('Significant_DEGs.csv', index=False)

# Further filter based on log2 fold change ≥ 1 (fold change ≥ 2)

significant_genes = results_df[

(results_df['Adjusted_P_Value'] < 0.05) &

(abs(results_df['Log2_FC']) >= np.log2(2))

]

print(f"Number of significant genes: {len(significant_genes)}")

significant_genes.to_csv('significant_genes.csv', index=False)

# Identify genes upregulated in the high and low expression groups

high_expression_genes = significant_genes[significant_genes['Log2_FC'] > 0]

low_expression_genes = significant_genes[significant_genes['Log2_FC'] < 0]

# Save upregulated genes for each group

high_expression_genes.to_csv('tumor_expression_genes.csv', index=False)

low_expression_genes.to_csv('normal_expression_genes.csv', index=False)

# Output the number of genes in each group

print(f"Number of high expression genes: {len(high_expression_genes)}")

print(f"Number of low expression genes: {len(low_expression_genes)}")

# Create a volcano plot

plt.figure(figsize=(10, 7))

# Plot all genes in gray

plt.scatter(results_df['Log2_FC'], -np.log10(results_df['Adjusted_P_Value']), color='grey', alpha=0.5)

# Plot high expression genes in red

plt.scatter(high_expression_genes['Log2_FC'], -np.log10(high_expression_genes['Adjusted_P_Value']), color='red', alpha=0.8, label='High Expression')

# Plot low expression genes in blue

plt.scatter(low_expression_genes['Log2_FC'], -np.log10(low_expression_genes['Adjusted_P_Value']), color='blue', alpha=0.8, label='Low Expression')

# Axis labels

plt.xlabel('Log2 Fold Change', fontsize=13)

plt.ylabel('-log10(Adjusted P-Value)', fontsize=13)

plt.xticks(fontsize=12)

plt.yticks(fontsize=12)

# Set axis limits

plt.xlim(-6, 6)

plt.ylim(0, 20)

# Threshold lines

plt.axhline(y=-np.log10(0.05), color='black', linestyle='--', label='P-Value = 0.05')

plt.axvline(x=np.log2(2), color='black', linestyle='--', label='Fold Change = 2')

plt.axvline(x=-np.log2(2), color='black', linestyle='--')

# Show legend and plot

plt.legend()

plt.tight_layout()

plt.show()

1. **Python cord for SVM**

import pandas as pd

from sklearn.model_selection import train_test_split

from sklearn.svm import SVC

from sklearn.metrics import (

accuracy_score, confusion_matrix, classification_report,

roc_curve, auc, ConfusionMatrixDisplay

)

from sklearn.preprocessing import StandardScaler

import matplotlib.pyplot as plt

# Load dataset and remove missing values

df = pd.read_csv('Nectin 4 immnoscore 3.csv')

df.dropna(inplace=True)

# Separate features and target variable

X = df[['Nectin4 et al. IS', 'Age', 'Sex', 'right=0', 'renal 2', 'Tumor morphology', 'Tumor grade', 'pT stage']]

y = df['Death'] # Target variable indicating death status

# Standardize features

scaler = StandardScaler()

X_scaled = scaler.fit_transform(X)

# Split into training and test datasets

X_train, X_test, y_train, y_test = train_test_split(

X_scaled, y, test_size=0.6, random_state=42)

# Train a Support Vector Machine with linear kernel and probability estimates

clf = SVC(kernel='linear', probability=True, class_weight='balanced', random_state=42)

clf.fit(X_train, y_train)

# Predict labels

y_pred_test = clf.predict(X_test)

y_pred_train = clf.predict(X_train)

# Obtain probability scores for ROC analysis

y_prob_test = clf.predict_proba(X_test)[:, 1]

y_prob_train = clf.predict_proba(X_train)[:, 1]

# Compute ROC curves and AUC scores

fpr_test, tpr_test, _ = roc_curve(y_test, y_prob_test)

roc_auc_test = auc(fpr_test, tpr_test)

fpr_train, tpr_train, _ = roc_curve(y_train, y_prob_train)

roc_auc_train = auc(fpr_train, tpr_train)

# Extract weighted average metrics from classification report

report_test = classification_report(y_test, y_pred_test, output_dict=True)

report_train = classification_report(y_train, y_pred_train, output_dict=True)

wp_test = report_test['weighted avg']['precision']

wr_test = report_test['weighted avg']['recall']

wf_test = report_test['weighted avg']['f1-score']

wp_train = report_train['weighted avg']['precision']

wr_train = report_train['weighted avg']['recall']

wf_train = report_train['weighted avg']['f1-score']

# Plot ROC curves

plt.figure()

plt.plot(fpr_test, tpr_test, color='darkorange', lw=2,

label='Test AUC: %.2f\nPrecision/Recall/F1-score: %.2f, %.2f, %.2f' %

(roc_auc_test, wp_test, wr_test, wf_test))

plt.plot(fpr_train, tpr_train, color='blue', lw=2,

label='Train AUC: %.2f\nPrecision/Recall/F1-score: %.2f, %.2f, %.2f' %

(roc_auc_train, wp_train, wr_train, wf_train))

plt.plot([0, 1], [0, 1], color='gray', lw=2, linestyle='--')

# Format plot

plt.xlim([0.0, 1.0])

plt.ylim([0.0, 1.05])

plt.xlabel('False Positive Rate')

plt.ylabel('True Positive Rate')

plt.legend(loc="lower right", fontsize=10)

plt.grid(True)

# Save the figure as a high-resolution image file

plt.savefig('roc_curve.png', dpi=300)

plt.show()

1. **Python cord for Random Forest**

import pandas as pd

from sklearn.model_selection import train_test_split

from sklearn.ensemble import RandomForestClassifier

from sklearn.metrics import (

classification_report, roc_curve, auc

)

from sklearn.preprocessing import StandardScaler

import matplotlib.pyplot as plt

# Load dataset and remove missing values

df = pd.read_csv('Nectin 4 immnoscore 3.csv')

df.dropna(inplace=True)

# Define features and target variable

X = df[['Nectin4 et al. IS', 'Age', 'Sex', 'right=0', 'renal 2', 'Tumor morphology', 'Tumor grade', 'pT stage']]

y = df['Death'] # Target variable indicating death status

# Standardize feature values

scaler = StandardScaler()

X_scaled = scaler.fit_transform(X)

# Split into training and test datasets while maintaining class balance

X_train, X_test, y_train, y_test = train_test_split(

X_scaled, y, test_size=0.6, random_state=42, stratify=y)

# Train a Random Forest classifier with class balancing

clf = RandomForestClassifier(n_estimators=100, class_weight='balanced', random_state=42)

clf.fit(X_train, y_train)

# Predict class labels

y_pred_test = clf.predict(X_test)

y_pred_train = clf.predict(X_train)

# Predict class probabilities for ROC analysis

y_prob_test = clf.predict_proba(X_test)[:, 1]

y_prob_train = clf.predict_proba(X_train)[:, 1]

# Compute ROC curves and AUC values

fpr_test, tpr_test, _ = roc_curve(y_test, y_prob_test)

roc_auc_test = auc(fpr_test, tpr_test)

fpr_train, tpr_train, _ = roc_curve(y_train, y_prob_train)

roc_auc_train = auc(fpr_train, tpr_train)

# Extract weighted average metrics from classification reports

report_test = classification_report(y_test, y_pred_test, output_dict=True)

report_train = classification_report(y_train, y_pred_train, output_dict=True)

wp_test = report_test['weighted avg']['precision']

wr_test = report_test['weighted avg']['recall']

wf_test = report_test['weighted avg']['f1-score']

wp_train = report_train['weighted avg']['precision']

wr_train = report_train['weighted avg']['recall']

wf_train = report_train['weighted avg']['f1-score']

# Plot ROC curves

plt.figure()

plt.plot(fpr_test, tpr_test, color='darkorange', lw=2,

label='Test AUC: %.2f\nPrecision/Recall/F1-score: %.2f, %.2f, %.2f' %

(roc_auc_test, wp_test, wr_test, wf_test))

plt.plot(fpr_train, tpr_train, color='blue', lw=2,

label='Train AUC: %.2f\nPrecision/Recall/F1-score: %.2f, %.2f, %.2f' %

(roc_auc_train, wp_train, wr_train, wf_train))

plt.plot([0, 1], [0, 1], color='gray', lw=2, linestyle='--')

# Format the plot

plt.xlim([0.0, 1.0])

plt.ylim([0.0, 1.05])

plt.xlabel('False Positive Rate')

plt.ylabel('True Positive Rate')

plt.legend(loc="lower right", fontsize=10)

plt.grid(True)

# Save the plot as a high-resolution image

plt.savefig('roc_curve.png', dpi=300)

plt.show()

1. **Python cord for correlation analysis**

import numpy as np

import pandas as pd

import matplotlib.pyplot as plt

import seaborn as sns

# Load the dataset

df_score = pd.read_csv("Subtype marker.csv", index_col=0)

# Calculate the correlation matrix

df_corr = df_score.corr()

print(df_corr)

# Generate a heatmap of the correlation matrix

plt.figure(figsize=(12, 10))

sns.heatmap(

df_corr, # Correlation matrix to visualize

vmin=-1, vmax=1, # Fixed scale range for consistent color representation

annot=True, fmt='0.2f', # Display correlation coefficients with two decimal places

cmap=sns.color_palette('coolwarm', 100), # Color gradient from cool to warm

# square=True, # Optional: enforce square cells

)

# Display the heatmap

plt.show()
